# Role of Oral Antioxidant Supplementation in the Current Management of Diabetic Retinopathy

**DOI:** 10.3390/ijms22084020

**Published:** 2021-04-13

**Authors:** Enrique Antonio Alfonso-Muñoz, Raquel Burggraaf-Sánchez de las Matas, Jorge Mataix Boronat, Julio César Molina Martín, Carmen Desco

**Affiliations:** 1 FISABIO-Oftalmología Médica (FOM), Vitreoretinal Unit, 46015 Valencia, Spain; enalmuo@gmail.com (E.A.A.-M.); jorge.mataix@gmail.com (J.M.B.); 2 Servicio de Oftalmología, Hospital General Universitario de Castellón, 12004 Castellón, Spain; raquelburggraaf@gmail.com; 3 Servicio de Oftalmología, Hospital Universitario San Juan, 03550 Alicante, Spain; jcmm.molina@gmail.com

**Keywords:** diabetic retinopathy, antioxidants, oxidative stress, management, clinical practice, ophthalmology, retina, nutrition, nutraceuticals, multivitamin complex

## Abstract

Oxidative stress has been postulated as an underlying pathophysiologic mechanism of diabetic retinopathy (DR), the main cause of avoidable blindness in working-aged people. This review addressed the current daily clinical practice of DR and the role of antioxidants in this practice. A systematic review of the studies on antioxidant supplementation in DR patients was presented. Fifteen studies accomplished the inclusion criteria. The analysis of these studies concluded that antioxidant supplementation has a IIB level of recommendation in adult Type 1 and Type 2 diabetes mellitus subjects without retinopathy or mild-to-moderate nonproliferative DR without diabetic macular oedema as a complementary therapy together with standard medical care.

## 1. Introduction

Diabetic retinopathy (DR) is one of the most specific microvascular complications in diabetes mellitus (DM) and is the main cause of avoidable blindness in working-aged people [1,2,3]. As it develops, and depending on the duration of DM and glycaemic levels, it is expected to increase concurrently as diabetes numbers rise [1]. According to the most recent International Diabetes Federation data, the worldwide prevalence of DM was estimated to be 9.3% (463 million people) in adults aged 20–79 years old in 2019, and these numbers are expected to increase to 10.9% (700.2 million people) by 2045, thereby constituting a global burden for the Public Health Care Systems that will not stop increasing [2].

One of the main concerns is that patients can remain asymptomatic until visual symptoms begin. The current management of DR comprises accurate screening programmes for early detection and a strict systemic control of glycaemia and related risk factors; however, active treatment is not initiated until advanced stages of the disease occur [3]. Taking into account that up to 45.8% of the world diabetic population is estimated to be undiagnosed, many are checked once DM complications have already developed, when tissue damage may be irreversible [4]. There is still a gap for new diagnostic biomarkers and for active treatments that could be applied in the early stages of DR to prevent further progression.

Oxidative stress has been demonstrated to play a key role in the pathophysiology of DR and has been postulated as a nexus with other biochemical pathways, which commonly brings about inflammation, neurodegeneration, and microvasculopathy [5,6]. The use of antioxidants, such as polyphenols, has proved to reduce DR progression in animal models [7]. However, their role in the clinical management of DR patients has not yet been established. We presented updated guidelines on DR followed by a systematic review of antioxidant clinical trials discussing when, how and why antioxidant supplementation should be used in DR management.

## 2. Epidemiology and Risk Factors

According to the latest published report by the Vision Loss Expert Group in 2015, DR dropped from fifth to sixth place of the most common cause of preventable vision impairment globally [8], in comparison with their previous report in 2010 [9]. Nevertheless, while blindness and vision impairment due to all causes has decreased, DR was the only eye disease that had risen in terms of crude global prevalence since 1990: DR-blindness increased by 7.7% and DR moderate-to-severe vision impairment increased by 28.6% in 2015. This was a warning signal to strengthen existing screening programmes and management strategies and increase research programmes directed towards new molecular targets before the disease appears [8,10].

In 2010, the global prevalence of DR among the diabetic population was estimated to be about 34.6% with regard to DR of any kind, 6.96% for proliferative diabetic retinopathy (PDR), 6.81% for diabetic macular oedema (DME), and 10.2% for vision-threatening diabetic retinopathy (VTDR), including PDR and DME. This means that approximately one third of DM patients would have some degree of DR and one tenth needs active treatment. The risk of having some kind of DR was 2.7 times higher in type 1 DM (T1DM) than in type 2 DM (T2DM) after at least 20 years of disease progression [1].

Regarding DR global incidence, a recent nine-year prospective population-based study including T1 and T2 patients determined an annual incidence of 15.16 ± 2.19% in T1DM, in comparison to 8.37 ± 2.19% in T2DM. In both the duration of DM and glycaemic control, together with arterial hypertension, low-density lipoprotein (LDL) cholesterol and creatinine levels were significant risk factors for developing DR, both in T1DM and T2DM. A higher prevalence and incidence of DR observed in the T1DM sample at the end of this study was attributed to a longer duration of diabetes and worse basal and monitored HbA1c levels [11].

In the context of dietary patterns, there is evidence that dietary fibre, oily fish and the Mediterranean diet can protect against DR. By contrast, a higher caloric intake has been associated with a greater risk for DR. In addition, a recent review reported that studies on antioxidants, specifically vitamins C, E and carotenoids as protective factors in usual diets, are inconclusive [12].

## 3. Pathophysiology

Diabetic retinopathy must be considered as multifactorial, as its development is a direct consequence of prolonged levels of hyperglycaemia, although it is influenced by the previously mentioned risk factors [1,11,13,14].

From a metabolic perspective, an exacerbated oxidative stress environment has been found to be a crucial factor in the development of DR, interacting as a link between other glucose-mediated biochemical processes. An excess of glucose causes the activation of the polyol pathway, the production of advanced glycation end products (AGEs), the activation of protein kinase C (PKC) and an increase in the hexosamine pathway. All together, these contribute to an increase in the production of reactive oxygen species (ROS) [5,6,15]. Although these molecules are continuously produced for normal cell function, they are neutralised through physiological antioxidant systems. Moreover, ROS accumulation triggers structural changes concerning mitochondrial DNA and the subsequent modifications in gene expression. The impact of oxidative stress on diabetes is not temporary, because mitochondrial dysfunction involves the exacerbation of the production of ROS [16,17]. This occurrence, described as the metabolic memory, has been postulated to be responsible for disease progression even after achieving correct glycaemic control [18]. Among the cited risk factors for DR, arterial hypertension has been demonstrated to increase glycaemic-induced oxidative stress, contributing to the exacerbation of molecular damage [19].

Metabolic alterations produced by hyperglycaemia and an increase in ROS result in cellular dysfunction and sequential apoptosis, leading to [20]:

- Inflammation: Microglial activation seems to be an early event in DR and can trigger the secretion of inflammatory mediators [21,22]. Proinflammatory cytokines (interleukins IL-1β, IL-6, IL-8 and TNF-α) have been reported in higher levels in human vitreous samples [23,24], and have also correlated with the severity of DR [25]. Under inflammatory stimuli, endothelial cells increase the expression of intracellular and vascular adhesion molecules (ICAM-1 and VCAM-1) and E-selectin, allowing leukocyte adhesion to the endothelial cell walls and the production of leukostasis, which is a determining factor for posterior microvascular damage [26,27]. Increased levels of the above-mentioned molecules have been reported in diabetic blood samples [28], and the inhibition of ICAM-1 in cultured human retinal endothelial cells from diabetic patients reduced cell apoptosis. Interestingly, the use of an antioxidant agent reduced the levels of ICAM-1 on those retinal cultures and was able to reduce cellular loss [29].
- Neurodegeneration: Apoptosis seems to affect neurons before vascular cells. Electroretinogram (ERG) studies have shown the possibility of existing neuronal damage even before DR clinical signs were present, preceding microvascular changes. Furthermore, retinal analysis has demonstrated a thinner ganglion cell inner layer both in diabetic animal models and human subjects [30,31,32]. With regard to the role of oxidative stress in relation to neurodegeneration, ERG studies have been carried out on induced-diabetes mice, before and after antioxidant administration, thus verifying the protective role of lutein both on visual function and histological neuronal changes [31].
- Microvasculopathy: The walls of retinal capillaries have an external pericytes layer, a basement membrane and an inner endothelial cells layer. Pericyte loss occurs under hyperglycaemic conditions [33] and leads to focal microvascular dilatation with microaneurysm formation. A thickening of the basement membrane, due to debris deposition and endothelial cell dysfunction, leads to blood–retinal barrier (BRB) disruption, producing increased vascular permeability with exudation and haemorrhages [34]. Leukostasis derived from an inflammatory response is involved in endothelial cell impairment and is followed by microvascular occlusions [27]. Subsequent hypoxia promotes the activation of transcription factor hypoxia-inducible factor 1 (HIF-1), which further stimulates the secretion of vascular endothelial growth factors (VEGFs), and therefore causes neovessel formation [35].

## 4. Important Role of Early Diagnosis

### 4.1. Classification of DR

Fundoscopy clinical signs derived from tissue damage have suggested a sequential grading of DR. In 2002, in an attempt to simplify the daily clinical practice, the Global Diabetic Retinopathy Project Group proposed the International Clinical Disease Severity Scale for DR, as shown in Table 1 [36].

A recent multicentre cohort study performed in the UK analysed the five-year risk of developing PDR depending on the stage at baseline. The probability of presenting PDR at year five was 2.2% for no initial DR, 13% for mild nonproliferative diabetic retinopathy (NPDR) at baseline, 27.2% for moderate NPDR and 45.5% for severe NPDR [37].

**Figure 1:**
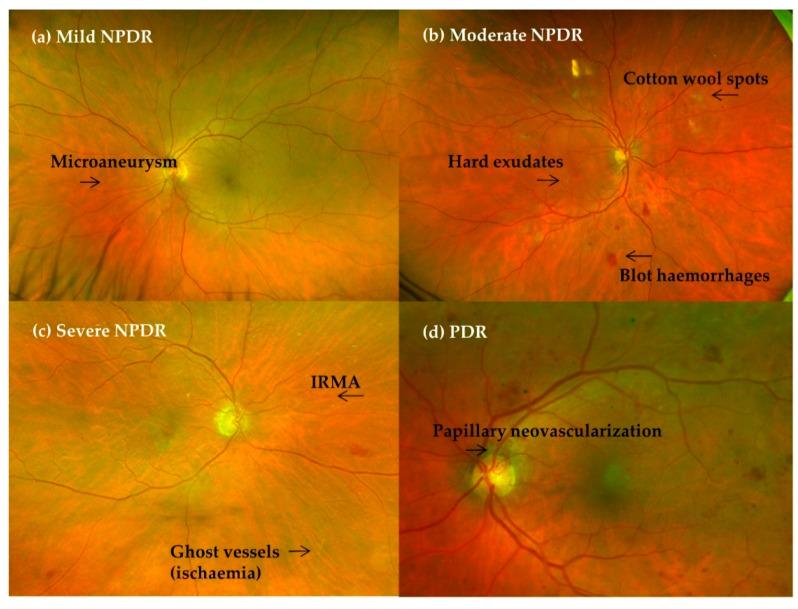
Clinical DR progression. (**a**) Mild nonproliferative diabetic retinopathy (NPDR), (**b**) moderate NPDR, (**c**) severe NPDR and (**d**) proliferative diabetic retinopathy (PDR) with papillary neovascularisation.

Diabetic macular oedema (DME) has been classified separately, as it can appear at any of the mentioned stages of DR. The International Scale understood DME to be the retinal thickening or presence of hard exudates in the posterior pole, and it was graded into three stages [23] (Table 1 and Figure 1).

### 4.2. Impact on Visual Impairment

Studies that have measured quality-of-life items reported vision-related effects on emotional well-being and general health, especially in those suffering from VTDR, contributing to a lack of economic development [38,39]. DME is the most common reason for visual acuity (VA) loss, followed by any context of PDR [1,11].

From the viewpoint of ophthalmological care, the active treatment of DR should be initiated when a high risk of progression to PDR or DME [3] is evident. It must be kept in mind that advanced stages of DR or DME may translate into extensive tissue damage with dysfunctional cells due to the metabolic memory effect [18,40,41] and, therefore, make it too late to restore adequate visual function.

## 5. Imaging Techniques for Diabetic Retinopathy Screening

The exponential increase in diabetes worldwide has forced the development of several imaging diagnostic tools that can screen for DR in a fast and effective way. Traditionally, most DR screening programmes have been based on fundus photography (FF) with mydriatic or nonmydriatic cameras to identify DR patterns. Nowadays, imaging technology has made a significant quantitative and qualitative leap forward, providing a great improvement in terms of affordability, performance and diagnostic accuracy. Funduscopic imaging using smartphones, macular optical coherence tomography (OCT) and ultrawide-field imaging, together with revolutionary artificial intelligence (AI) software, has opened the door to a new era in the early diagnosis and follow-up of DR patients.

The International Council of Ophthalmology (ICO) guidelines for DR diagnosis include retinal examination with either (a) direct or indirect ophthalmoscopy or fundus biomicroscopy using a slit lamp, or (b) mono-photography or stereo-photography greater than or equal to 30°, associated or not with OCT. These images should always be analysed and classified by a trained specialist [3]. The accuracy of DR classification provided by these screening tools is considered to be category 1, as defined by the American Teleophthalmological Association (ATA), and it provides a sufficiently high level of accuracy for population screening.

### 5.1. Fundus Photography

Today’s digital FF is a reliable alternative to the traditional seven-field photography used in early treatment diabetic retinopathy studies (ETDRSs) [42]. Current fundus cameras have resolutions in the range of 20 megapixels, exceeding the 2-3-megapixel resolution needed to display a single retinal microaneurysm [43]. Several clinical trials have shown that mydriatic FF has a higher sensitivity and specificity for DR detection compared to nonmydriatic cameras [43]. However, due to practical and logistical considerations, most DR detection programmes use nonmydriatic FF. Most modern programmes use a single 45° photograph or even two or three multi-field photographs [44].

The ATA establishes four levels of grading systems for DR. Level 1 includes programmes that can distinguish between the absence of DR and minimal DR disturbances. Level 4 can make a complete DR categorisation between mild, moderate, severe and early proliferative high-risk DR, with or without macular oedema [44].

### 5.2. Ultrawide-Field Imaging for Diabetic Retinopathy Screening

Wide-field photography is another DR screening modality, which is especially appropriate for detecting peripheral ischaemic lesions and proliferative diabetic retinopathy [45]. Ultrawide-field imaging reaches 80% of the retinal surface and detects DR with a 99% sensitivity and 97% specificity [46].

### 5.3. Optical Coherence Tomography for Diabetic Macular Oedema Screening

As mentioned above, FF is a useful diagnostic tool for DR, but it has a low accuracy in detecting diabetic macular oedema (DME) due to its two-dimensional nature and because it is unable to measure retinal thicknesses reliably. The English National Screening Program for the detection of DME uses three photographic landmarks: first, the appearance of exudates within one disc diameter from the fovea; second, the presence of circinate exudates within the macula; and third, the presence of microaneurysms or haemorrhages within one disc diameter from the fovea [47]. However, this method yields limited diagnostic utility. Conventional FF offered an 86.6% false positive rate in detecting DME, according to a cross-sectional observational study [48]. For this reason, macular OCT is a better option than FF for evaluating the macula. Several studies have investigated the cost-effectiveness and efficiency of using macular OCT in DME detection programmes [49].

OCT allows for a detailed analysis of all the retinal layers. For example, it quantifies the thinning of the ganglion cell and nerve fibre layer due to the neurodegenerative process present in DR patients. It may also show the disorganisation at the macular internal retinal layer (DRIL) as a biomarker of visual impairment [50]. Alternatively, it may evaluate the integrity of the outer layers where the cone photoreceptors and the pigment epithelium cells of the retina are present.

### 5.4. OCT Angiography in Diabetic Retinopathy Screening

OCT angiography (OCT-A) is a novel noninvasive imaging modality able to describe the retinal circulation using algorithms that detect red blood cell movement. This technique can detect microaneurysms, retinal neovascularisation or ischaemic areas, but only in the posterior pole; it cannot evaluate the peripheral retina [51]. Therefore, it can classify DR according to its severity. It describes the anatomy and blood flow at the foveal avascular zone and the superficial and deep retinal plexus in the macula [51,52]. OCT-A can also measure the progression of DR as it correlates vascular changes with visual acuity [53].

### 5.5. Smartphone Function in Diabetic Retinopathy Screening

Most digital FF cameras and OCTs are expensive and require trained specialists in retinal image interpretation. To solve this problem, several smartphone applications have been developed as effective and accessible techniques for DR detection. Cameras in modern cell phones have an even higher resolution than other conventional fundus cameras. New-generation smartphones have wide-field digital cameras with resolutions greater than 10 megapixels [44]. Currently, these smartphones need the coupling of supplementary lenses and specific image editing software. Several clinical trials have demonstrated the potential of these devices to detect DR in different regions of the world [54].

### 5.6. Automated DR Image Evaluation Systems Used for Teleophthalmology

Gardner and colleagues described the use of an artificial neural network able to detect DR with an 88% and 83% sensitivity and specificity, respectively, as compared to a trained ophthalmologist [55]. These systems use pattern recognition algorithms that identify specific features of DR, such as microaneurysms. On the downside, the image pre-processing requires a lot of time in order to identify clinically important features and develop mathematical descriptors for the different types of lesions.

Deep learning (DL) is a novel AI iteration that employs convolutional neural networks to interpret images by repetitive analysis and comparison of the output with a standard that is self-correcting if an error is made. Multiple studies have demonstrated useful results in the development of DL algorithms that are able of identify references of DR without the need to previously teach computer systems specific DR characteristics. The sensitivity and specificity of these approaches are generally 90% and 95%, respectively [56,57]. The present generation of DL algorithms employing coloured FF has achieved a rating performance similar to that of a retinal specialist [58]. Several DL algorithms for DR detection, including the recently FDA-approved IDx-DR algorithm [59], have been described. These systems are trained using known features of DR, such as microaneurysms, bleeding and exudates.

## 6. Pathology Control and Monitoring

The correct management of a disease usually depends on the good practice of doctors, their diagnosis and application of the appropriate treatment. However, controlling a high-prevalence disease, such as DR, which affects almost 100 million people worldwide [1], needs more than diagnosis and treatment; it requires good follow-up protocols in order to optimise health resources and focus attention on patients who are more susceptible to having vision loss complications.

### 6.1. First Visit

Knowing when to first examine a diabetic patient for DR signs is the first step in controlling and preventing the pathology.

Guidelines differ: UK guidelines recommend to start DR screening in all persons diagnosed with diabetes aged 12 and over [60] as a result of research including 2125 children under 12-years-old diagnosed with DM, showing that only three children (0.17%) presented sight-threatening conditions, and none required treatment [61]. The American Academy of Ophthalmology Guideline recommends to start screening five years after the diagnosis of T1DM and when T2DM is diagnosed [62].

The first visit is most important and should incorporate the following crucial actions:

#### 6.1.1. Grade the Diabetic Retinopathy

The main goal of ophthalmologists and general doctors treating DR patients is to avoid vision loss complications of DR, diabetic macular oedema (DME) and proliferative diabetic retinopathy (PDR). In order to plan a good strategy to prevent these complications, DR has been divided into different grades depending on the probability of developing these complications and the guidance for their management.

Following the International Classification of Diabetic Retinopathy and Diabetic Macular Edema [3], the risk of developing PDR and the proposed follow-up time in each DR grade are listed in Table 2.

**Figure 2:**
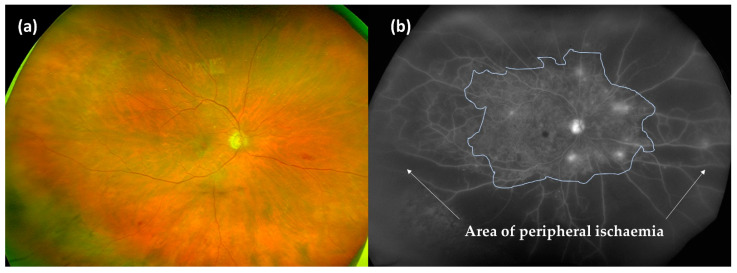
Wide-field photography (**a**) and its correspondent wide-field fluorescein angiography (WF-FA) (**b**). Surprisingly, the ischaemic areas (white arrows) in WF-FA are much larger than was suspected with funduscopy findings, which clearly changes the DR grading.

**Figure 3:**
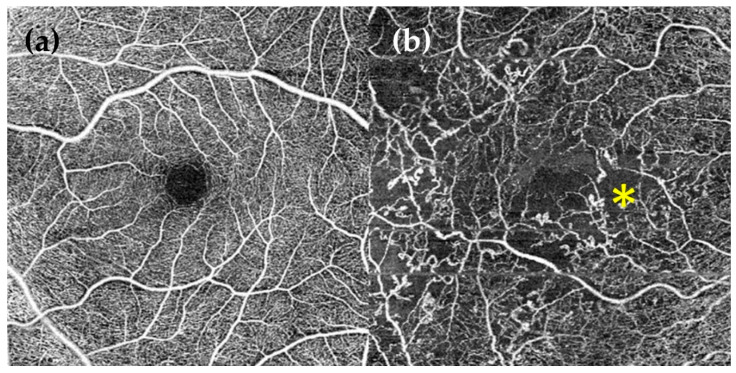
Optical coherence tomography angiography (OCT-A) of 6 × 6 mm. (**a**) Healthy eye. Normal foveal avascular zone (FAZ); (**b**) DR eye. Abnormal changes in FAZ size and shape. Microaneurysms (*), vessels loops, tortuosity and a reduction of capillary density can be observed.

Apart from a normal ophthalmology examination including funduscopy, other important complementary explorations to grade DR at the first visit are strongly recommended:

- Wide-field fluorescein angiography (WF-FA): Though some guidelines recommend performing FA on a case-by-case basis prior to macular laser treatment [60], analysing peripheral retina vascularisation is fundamental for DR management as global ischaemia is related to neovascularisation, which indicates treatment to prevent PDR [63]. As wide-field OCT angiography (WF-OCTA) is as yet uncommon, our group recommend performing a WF-FA at a moderate NPDR stage. WF-FA is also, to date, the best exploration for grading DR [64], detecting 1.6 to 3.5-fold more fields affecting DR severity than ultrawide-field colour imaging (Figure 2).
- Optical coherence tomography (OCT): OCT should be mandatory at every DR visit as several DMEs can only be seen in OCT examination. OCT is also the most useful imaging modality for calculating and monitoring the individual treatment response to anti-VEGF treatment [65].
- OCT angiography (OCT-A): OCT-A can demonstrate areas of capillary nonperfusion and it is very useful for assessing patients with DR and loss of visual acuity without central oedema. An increase in the area of the foveal avascular zone has been associated with worse visual acuity [60,66,67,68] (Figure 3).

#### 6.1.2. Educational Advice

Once the DR of the patient has been graded, educational advice on the first visit is fundamental to encourage patients to attend their routine appointments. A retrospective audit of data from 62,067 patients due for annual diabetes eye screening showed that missing appointments strongly increased the proportion of patients showing referable retinopathy at the next visit. Particular focus must be put on young T1DM male patients who are the most likely to miss appointments [69]. At this point, general practitioners, endocrinologists and ophthalmologists should work together throughout the whole follow-up.

A proposed scheme of the advice is:

DR is the first cause of blindness in working-age people [8]. DR is a reflection of systemic affection by diabetes, and in fact, the presence of mild NPDR implies an excess of mortality of 81% and a moderate-severe NPDR of 314% [70]. As a result, uncontrolled DR is a reflection of uncontrolled diabetes, increasing possibilities of not only blindness, but also death due to some other cause. However, blindness caused by DR is almost 100% avoidable following our recommendations [71].

#### 6.1.3. Establishing Goals for Controlling the Risk Factors Associated with DR Progression

While DR is in its early stages, the risk of losing vision is low and controlling the risk factors associated with the development and progression of DR is the only treatment we can offer patients. Specific goal values have to be established. The main goals for DR control are to regulate HbA1c and blood pressure (BP). Secondary goals include lowering serum lipids and obesity.

- HbA1c < 7%: HbA1c control has a memory effect, in other words, the effect of the correct control over time protects against the progression of DR in the case of a future uncontrolled period [72]. Therefore, the early intervention in this parameter is essential. A 1% reduction in HbA1c is associated with a 35% reduction in the risk of developing DR, 15–25% in its progression, 25% in VA loss and 15% in developing blindness [73]. In T1DM compared to HbA1c of 9%, HbA1c under 7% diminishes the development of DR in 75% of cases and progression in 50%. Despite the importance of diminishing HbA1c, this reduction should not be acute, because, apart from the risk of hypoglycaemia, this reduction could promote DR progression, as shown in a study focusing on obese patients who underwent bariatric surgery. Notably, 18.9% of the patients who did not have DR before surgery developed DR in the first year after the procedure [74]. A DR study by an ophthalmologist is recommended before bariatric surgery. The goal of a HbA1c under 7% is variable depending on the patient [75]. A value under 6.5% is recommended if there is a risk of nephropathy and DR. A value between 7.1 and 8.5% could be tolerated if there are multiple comorbidities and if, despite maximum treatment, it is difficult to reach a value below 7%.
- BP < 150/85: When comparing patients with BPs under 180/10 mmHg with patients under 150/85 mmHg, there is a 33% reduction in the progression of DR and the necessity of laser treatment and a 50% reduction in vision loss in patients with lower BPs [76]. Although these data are classic, two more modern reviews and a meta-analysis indicate that reducing BP prevents the development of DR for up to four to five years [77] and reduces the relative risk of incidence of DR by 17% [78], but there is no clear evidence on slowing the progression once the disease has developed. In contrast to glycaemic control, BP control does not have a memory effect; once it becomes decompensated, the risk of progression of the disease increases regardless of the previous control.
- Lipid control: Two randomised control trials (RCTs) have independently demonstrated that fenofibrate reduces DR progression in T2DM and the need for laser treatment [79,80].
- Obesity: A meta-analysis published in 2018 showed that obesity increased DR with a relative risk of 1.2, more in T2DM. Obesity was not associated with PDR [81].
- Smoking: The association of smoking and DR has been established for T1DM, but not for T2DM [82].

Controlling these risk factors in DR patients by means of antioxidant supplementation is discussed further in the antioxidant review.

### 6.2. Follow-Ups

Follow-ups should be scheduled in accordance with the recommendations of the International Classification of Diabetic Retinopathy and Diabetic Macular Edema [3]. In follow-up visits, every patient should be regraded by exploring their visual acuity, fundus, and periphery of the retina, and by performing an OCT. The following visit should be programmed depending on the new grade of the DR. It is necessary to go over the educational advice.

**Figure 4:**
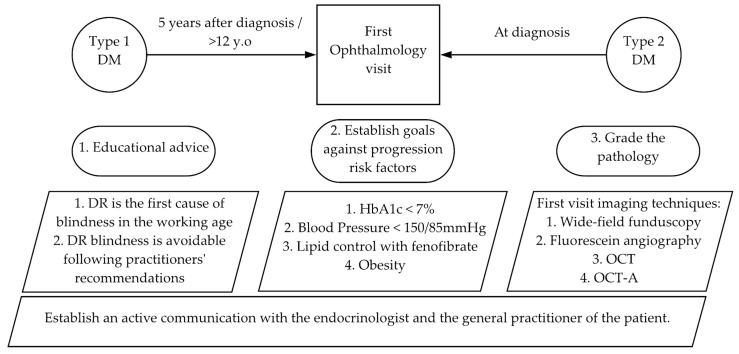
The first ophthalmology visit of a DR patient.

A general scheme of the first ophthalmology visit and the management of the disease is presented in Figure 4 and Table 2.

### 6.3. Consideration of Special Situations

#### 6.3.1. DR and Pregnancy

DM is estimated to affect 17% of pregnancies worldwide [83]. The majority suffer from gestational DM, which is not associated with DR, but cases of undiagnosed T2DM may be detected during pregnancy, and this group could develop DR during or after pregnancy. DR is present in 14% of T2DM pregnant women [84] and between 34% and 72% of T1DM [85,86] pregnant women. As pregnancy increases the short-term risk of DR progression [86], pregnancy is recommended to be planned earlier, especially in T1DM, and screening during this period should be optimised.

Ophthalmologic explorations should be carried out before pregnancy and at the 28th week of amenorrhea (WA). If the pregnant woman has already been diagnosed with DR, an additional examination should be performed at 16 to 20 WA.

During the postnatal period, retinopathy that progresses during pregnancy usually tends to regress [87]. Screening should be advised up to 12 months if retinopathy has progressed during the third trimester.

If the patient has PDR, panretinal laser photocoagulation (PRP), ideally before the commencement of pregnancy, is recommended [88,89].

In the case of progressive DME needing treatment, the intravitreal injection of steroids is the best option [90,91,92], and no side effects in pregnancy have been reported.

A recent randomised controlled trial addressing antioxidant supplementation during pregnancy and diabetes showed that magnesium-zinc-calcium-vitamin D co-supplementation for six weeks in women with gestational diabetes reduced biomarkers of inflammation and oxidative stress, such as serum C-reactive protein and plasma malondialdehyde concentrations. A decreasing trend in the weight of newborns and the rate of macrosomia was also observed [93].

#### 6.3.2. DR and Cataract

DR and DME progress faster after cataract surgery, meaning that screenings should be increased after the operation [94]. The rate of developing treatment-requiring DME increases sharply in the year after cataract surgery for all grades of retinopathy, peaking in the 3–6 months’ postoperative period [95]. It is strongly recommended to stabilise DR and DME before surgery [96].

In cases where funduscopy is not possible, because of cataract opacity, it has to be evaluated as soon as possible after surgery in order to apply the correct treatment.

Adding an intravitreal injection of bevacizumab or triamcinolone to reduce the risk of DR progression is recommended during or after cataract surgery in patients with DR [97,98,99]. With the advent of PRP in the 1970s, the risk of severe vision loss from PDR was reduced by more than 90%. The Diabetic Retinopathy Study (DRS) showed that PRP reduced the risk of severe vision loss in eyes affected with PDR [100,101].

This review could find no study on the effect of antioxidant supplementation for the prevention of DME after cataract surgery in diabetic patients. A study in this direction could be of interest as DME is a high-burden cataract complication in these patients.

## 7. Current Medical Treatment and Future Therapeutic Approaches

### 7.1. Nonsevere Proliferative Diabetic Retinopathy. Anti-VEGF, PRP or Both?

**Figure 5:**
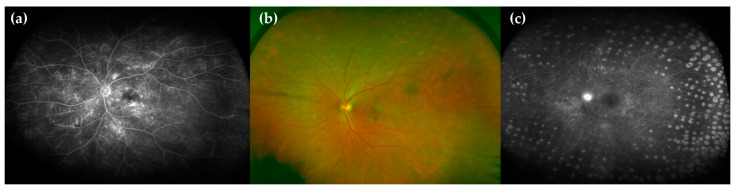
(**a**) Wide-field (WF)-Fluorescein angiography (FA) showing peripheral ischaemia and a generalised exudation. (**b**) Wide-field funduscopy showing laser spots after a panretinal photocoagulation (PRP) procedure. (**c**) No exudation is observed in the WF-FA after the PRP procedure, meaning pathology control.

Panretinal laser photocoagulation is effective in preserving central vision but can be associated with an exacerbation of macular oedema, loss of visual field, impaired night vision and loss of contrast sensitivity. Nevertheless, laser PRP has been considered the mainstay of treatment for PDR for a long time (Figure 5).

**Figure 6:**
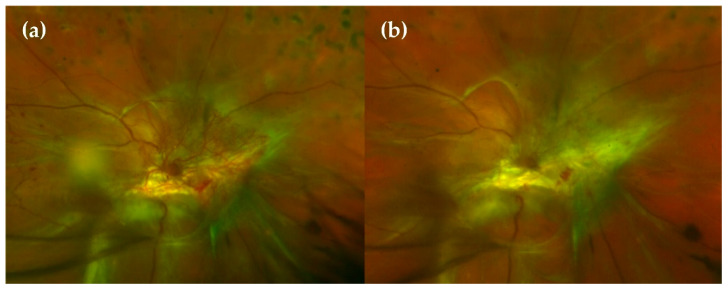
Anti-VEGF injection effect. (**a**) Papillary neovascularisation in a PDR eye before an anti-VEGF injection. (**b**) Clear regression of neovessels 3 days after the anti-VEGF injection.

It is known that high VEGF concentrations in the posterior segment of the eye are involved in the development of diabetic retinopathy and DME [102,103]. Anti-VEGF therapy for patients with DME showed a rapid regression of retinal neovascularisation and has made anti-VEGF therapy an alternative treatment for PDR, as shown in Figure 6. There has been increasing evidence from clinical trials that demonstrates that anti-VEGF injections are a safe and effective treatment for PDR over at least two years. The Diabetic Retinopathy Clinical Research (DRCR) Network and CLARITY studies show that there was no statistically significant visual acuity difference between the anti-VEGF (Ranibizumab and Aflibercept) and PRP groups after two years [104,105]. Different studies showed a better visual acuity, lower incidence of DME, less visual field reduction, and lower number of vitrectomies due to complications secondary to retinopathy in patients treated with anti-VEGF vs. PRP, but the disadvantage is that it presents a shorter-lasting effect of the treatment and requires more visits [106].

In 2018, the PACORES study revealed a synergistic effect when combining PRP with anti-VEGF, and it concluded that the treatment with ranibizumab + PRP is more effective than PRP monotherapy for neovascularisation regression [107]. These results suggest that intravitreal anti-VEGF therapy may be a viable alternative or adjunct to PRP for the treatment of eyes with PDR over at least two years. However, in clinical practice, anti-VEGF therapy for PDR presents some barriers that hinder the correct management of the disease; follow-up frequency, number of anti-VEGF injections, treatment costs and patients’ preference must be considered in each case [108]. The five-year retention rate, despite maximal efforts in the DRCR Retina Network protocol, was only 66% [109]. In one large, retrospective cohort study of patients with PDR, the follow-up absence over a four-year period was 584 out of 2302 patients (25.4%) [110]. Eyes with PDR only treated with intravitreal anti-VEGF presented worse anatomic and functional outcomes after the lack of follow-up for more than six months compared with eyes treated with PRP [111].

Anti-VEGF therapy has not been shown to improve retinal perfusion and may not prevent the nonperfusion progression in a diabetes-related eye disease. After the cessation of anti-VEGF therapy, recurrent neovascularisation can cause significant loss of visual acuity due to tractional retinal detachment or neovascular glaucoma [112].

The use of anti-VEGF in monotherapy to treat PDR requires exhaustive monitoring. Performing PRP, especially on eyes with clear ischaemic areas, reduces VEGF release in a sustained and lasting way. The synergistic effect of the anti-VEGF and PRP combination favours neovascularisation regression. In the case of DME with PDR, monotherapy with anti-VEGF is a good option, once the neovascularisation has been stabilised; if a new treatment is required, a re-evaluation with anti-VEGF or PRP would be the appropriate procedure [113].

### 7.2. Clinically Significant Macular Oedema (CSME)

**Figure 7:**
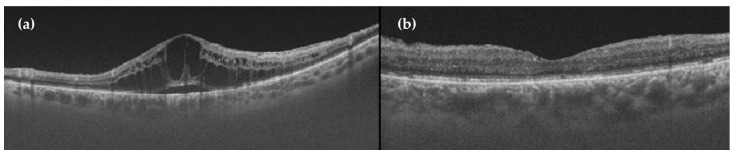
OCT showing the effect of anti-VEGF treatment in diabetic macular oedema (DME). (**a**) Cystic DME before anti-VEGF injection. (**b**) Normal macula 1 month after aflibercept (anti-VEGF) injection.

Focal laser photocoagulation has been the standard treatment for eyes with CSME. The early treatment diabetic retinopathy study (ETDRS) showed that focal or grid laser photocoagulation reduces the risk of further vision loss in patients with CSME and it has been proposed as a preferred treatment for CSME [114,115]. Intravitreal triamcinolone started to be used in the 2000s as a treatment for DME. In 2008, the DRCR Network showed that laser photocoagulation was superior to triamcinolone intravitreal therapy for DME treatment [116]. The “anti-VEGF age for DME treatment” began in 2010, when the DRCR Network showed that intravitreal injections of ranibizumab were better than laser for DME treatment [117]. Subsequently, several trials demonstrated that other anti-VEGF agents (bevacizumab and aflibercept) were also better than laser treatment (Figure 7). The FDA approved the use of aflibercept and ranibizumab as treatments for DME. Bevacizumab is used off-label for this condition. Intravitreal therapy with anti-VEGF is currently the standard of care in the management of eyes with central-involved diabetic macular oedema (CIDME). Numerous clinical trials have shown the advantage when compared with monotherapy or even combination therapy with laser [117,118,119,120,121,122]. Most eyes with DME respond to anti-VEGF therapy with some degree of anatomical and visual improvement, but in a significant number of eyes, the complete resolution of diabetic macular oedema is not achieved [123]. In clinical practice, an inadequate number of injections is relatively frequent during the follow-up, mainly due to the difficulty to adhere to monthly visits. A recent five-year follow-up study suggested that vision improved from baseline to five years without protocol-defined treatments after follow-up ended at two years, but vision worsened during the three years of standard care [124].

Intravitreal treatment with glucocorticoids for CIDME, such as sustained-release fluocinolone acetonide and dexamethasone implants, has been evaluated in multiple studies and has proved to be effective in reducing retinal thicknesses and in improving vision. However, intravitreal glucocorticoid treatment increases the risk of cataracts and glaucoma [125,126].

The European Society of Retinal Specialists does not recommend laser photocoagulation for the treatment of DME, and it suggests anti-VEGF treatment as a first-line therapy. Steroids have maintained a role in the management of chronically persistent DME [127].

Antioxidants may play an adjuvant effect to these treatments by down-regulating the expression of VEGF and pro-inflammatory pathways [128]. This concept was studied by Lafuente et al. who assessed the effectiveness of intravitreal ranibizumab combined with a dietary supplement rich in docosahexaenoic acid (DHA) and other antioxidants [129]. They found a significant improvement in the central subfield macular thickness compared to patients who only received ranibizumab. These results are discussed in the next section.

## 8. Role of Oral Antioxidant Supplementation

As shown in Table 2, observation and systemic risk factor control are the current treatments for DR patients until severe NPDR, PDR or DME develops. Consequently, physicians and researchers should focus their attention on preventing DR progression as the best tool for avoiding the DR-related loss of vision and blindness; in vitro and animal studies indicate that the pathophysiological pathways of DR, inflammation, neurodegeneration and vasculopathy could be alleviated by nutraceuticals [130].

It is postulated that antioxidants inhibit abnormal metabolism and slow DR progression by inhibiting the production of ROS, neutralising free radicals and augmenting the antioxidant defence system [10,131,132,133]. The role of oral antioxidants has provided promising results in other retinal diseases, such as age-related macular degeneration (AMD) [134,135], but studies with antioxidant supplements have not yet convinced the scientific community to include them as a routine treatment for DR patients.

Oral supplementation with natural antioxidants carries the great advantage of being a noninvasive treatment with presumably no harmful effects [130]. A pending challenge is to determine whether it is an effective treatment for diabetic retinopathy and to identify which agents would be most appropriate to treat these individuals.

The oxidation process starts with the generation of free radicals. The subsequent damage derives from the interaction of those molecules both with polyunsaturated fatty acids, essential elements of the cell membranes, but also with DNA, proteins and other lipids [136]. Depending on the mechanism of action, among antioxidants, we can distinguish enzymatic agents and nonenzymatic substances.

- Enzymatic antioxidants
  The enzymatic agents accomplish their antioxidant activity by disintegrating and removing free radicals. These are intrinsic intracytosolic enzymes (catalase, superoxide dismutase, glutathione peroxidase and peroxiredoxin) that carry chemical reactions in the presence of several cofactors, such as coenzyme Q10 (ubiquinone), copper (Cu), manganese (Mn), zinc (Zn) or selenium (Se). These cofactors have been added to oral supplementation to promote the inherent mechanisms of auto-defence in the cells [136,137,138].
- Nonenzymatic antioxidants
  The nonenzymatic agents act at a second level, disrupting the free radical chain reactions. The majority of them can be extracted from natural sources (plants and fruits), and the following categories are included within this group [130,136]:
  ◦ Vitamins: C, E and A.
  ◦ Polyphenols
    ▪ Flavonoids: large group of agents including flavonols, flavones, flavanones, flavanols (example: pycnogenol containing catechin and epicatechin), anthocyanins, isoflavonoids, homoisoflavonoids and chalcones.
    ▪ Nonflavonoids
      - Hydroxycinnamic acids: curcumin.
      - Stilbenes: resveratrol and pterostilbene.
  ◦ Carotenoids: lutein, zeaxanthin, crocin and crocetin.
- Others
  Alpha-lipoic acid [139], omega-3 polyunsaturated fatty acids [140], calcium dobesilate [141], Asiatic acid, extracts of Gingkgo biloba, turmeric root.
  Formulae containing different blends of the mentioned agents have been commercialised and used as in ophthalmologic pathologies [7,142,143]. Among them, Nutrof Omega^®^, Brudy Retina^®^, Diaberet^®^ and Vitalux Forte^®^ have been tested in patients suffering from DR. Divfuss^®^ was specifically developed for the Diabetes Visual Function Supplement Study [144]. Their composition and effects are discussed later. From them, Diaberet^®^ and Vitalux Forte^®^ are no longer available and their formulae have been updated to Visucomplex Plus^®^ and Vitalux Plus^®^ by the corresponding laboratories.
  Is it time to include oral antioxidants in the daily management of DR patients? In order to answer this question, a systematic review of studies on antioxidant oral supplementation in DR patients is presented.

### 8.1. Methods

As DR is a chronic pathology affecting millions of people around the world, only experimental, antioxidant supplementation studies on humans, with a follow-up of six or more months, comprising thirty or more DR patients (the value of normal distribution) have been included. Only studies in English, Spanish or French were included.

Inclusion and exclusion criteria, searches and excluded studies are presented in Supplementary File S1.

### 8.2. Results

After reviewing the abstracts of the 125 initial results by two different researchers, 15 studies were finally included: one case-control observational study [145] and 14 RCTs [129,144,146,147,148,149,150,151,152,153,154,155,156,157]. The studies included are described in Table 3.

Three of the 15 reviewed studies recruited T1DM patients [144,147,157], while 14 recruited T2DM individuals. The clinical profile varied from diabetics without retinopathy to diabetics with mild-to-moderate NPDR. None of the studies included severe NPDR or PDR. Five studies enlisted patients presenting DME [129,147,149,151,156]. Follow-up periods varied from six to sixty months.

The variables analysed were:

1. Clinical variables
  DR onset or progression
  DME onset or progression
  Best corrected visual acuity (BCVA) improvement
  Central macular thickness (CMT) changes
  Retinal nerve fiber layer (RNFL) thickness changes
  Retinal blood flow changes
  Number of Ranibizumab intravitreal injections required
2. Functional variables
  Retinal sensitivity (dB)
  Contrast sensitivity
  Glare sensitivity
  Macular pigment ocular density (MPOD)
3. Biochemical variables
  HbA1c% values
  High-density lipoprotein (HDL)/Low-density lipoprotein (LDL)/total cholesterol/triglyceride levels
  Lipid peroxidation products levels
  Plasma total antioxidant capacity
  ROS levels
  Interleukin 6 (IL-6) plasma levels
  Microalbuminuria
  Creatinine clearance

### 8.3. Discussion

#### 8.3.1. Clinical Variables Results

The clinical variables depended on subjective evaluation. Four studies analysed the influence of antioxidant supplementation on DR onset or the degree of DR progression [145,146,151,153]. Three of them found a significant delay in DR progression in patients receiving Nutrof Omega^®^ or grape seed proanthocyanidins extract (GSPE) supplementation, with follow-up periods of at least 12 months [145,146,151]. The group of Haritoglou et al. examined the onset of DME, basing the diagnosis on a funduscopic evaluation according to the ETDRS criteria, as an OCT device was not available at all the centres. They found no differences between supplemented patients and controls after 24 months of follow-up [154]. Finally, the group of Bursell et al. utilised fluorescein angiography to analyse the retinal blood flow in T1DM patients, and they observed a significant increase in the Vitamin E-supplemented group [157].

As for the quantitative variables, four of the eight trials evaluating CMT changes encountered a significant decrease in macular thickness, with follow-up periods ranging from 6 to 36 months, in patients supplemented with Crocin 15 mg [147], Brudyretina^®^ [129,149] and Diaberet^®^ tablets [152]. Nevertheless, it must be taken into account that the patients included in the studies by Lafuente et al. [129,149] received Ranibizumab injections and Brudyretina^®^ capsules simultaneously; consequently, the improvement cannot be directly assigned to the antioxidant administration.

Similarly, one study reported a significant reduction in the total RNFL thickness [151]. However, this change only affected left eyes and, therefore, it could be considered as a casual finding.

Despite these positive results, it is important to emphasise that none of the studies included in this review were able to prove significant changes in BCVA.

#### 8.3.2. Functional Variables Results

Quite a different scene is observed regarding the influence of antioxidants on retinal function. Three studies included the analysis of retinal sensitivity [144,156], contrast sensitivity [144,148], glare sensitivity [144] and/or MPOD [144], and all of them observed a significant improvement in patients supplemented with Lutein, DiVFuSS^®^ complex or C. Asiatica capsules. These changes were observed after relatively short follow-up periods, varying from six to fourteen months. The profile of the patients included by Zhang et al. and Chouss et al. was mild-to-moderate NPDR without CSME [144,148], while Forte et al. recruited T2DM patients with DME but without macular thickening in the OCT [156]. 

#### 8.3.3. Biochemical Variables Results

Only three of the ten studies analysing HbA1c% values reported an improvement [129,147,153] in supplemented patients. Concurrently, none of the studies could demonstrate significant changes in HDL/LDL/total cholesterol or triglyceride levels, nor were statistically significant results regarding microalbuminuria observed by Forte et al. [156]. Only Bursell et al. encountered an improvement in creatinine clearance in T1DM patients undergoing Vitamin E supplementation, but this effect was not maintained after a crossover period to placebo [157].

By contrast, promising numbers were observed regarding oxidation parameters. Among all the studies analysing antioxidant status [129,149,150,151,155], the changes reflected a significant reduction in lipid peroxidation end products and increased plasma total antioxidant capacity, in relation to Brudyretina^®^, Nutrof Omega^®^, Ubiquinone and Vitalux Forte^®^ supplementation, both in studies with the shortest follow-up periods (six months) and the longest (60 months). Domanico et al. [152] were also able to demonstrate decreased ROS levels in patients supplemented with Diaberet^®^.

As another interesting finding, Lafuente 2019 et al. [129] demonstrated a significant decrease in IL-6 plasma levels after 36 months of follow-up, suggesting an anti-inflammatory effect related to supplementation with DHA (Brudyretina^®^).

#### 8.3.4. Safety Profile

The majority of the studies reported no adverse events related to oral supplementation. Bursell et al. detected low thyroid hormone levels in relation to Vitamin E in one patient; such levels recovered after the discontinuation of the antioxidant [157]. Haritoglou et al. reported nutritional, vascular, cardiac and nervous system disorders, as well as infections, in 46% of the supplemented individuals vs. 48% in the placebo group [154]. From them, 10 vs. 4 patients reporting treatment-emergent adverse events were attributed to the trial treatment (whether α-lipoic acid or a placebo capsule), but its clinical nature was not specified and no mention about forced drug discontinuation is provided. In the study performed by Moon et al., 27% of the GSPE-supplemented subjects vs. 27.66% of the placebo group reported adverse reactions, which consisted of infections, gastrointestinal events, ocular and central nervous system disorders [146]. Gastrointestinal events were potentially associated with the treatment that was administered, and drugs were withdrawn in four patients of the supplemented group vs. one in the placebo group. Sanz-González et al. reported mild gastrointestinal discomfort related to Nutrof Omega^®^ treatment, but no rates are specified and supplementation was not withdrawn [145].

Studies analysing T1DM patients had a mean follow-up time ranging from six to eight months and no children were included. Before recommending nutraceuticals to children with T1DM, more research is needed on their potential adverse effects over both the short and long term [158].

#### 8.3.5. Limitations

Limitations of this systematic review include the high heterogeneity among studies, regarding the use of multiple antioxidants, the clinical variables studied and the follow-up periods, ranging from six to sixty months. Shorter follow-ups could explain the lack of significant results concerning clinical findings, given the chronic character of the disease.

Another considerable limitation would be the fact that some of the studies fail to assess administered drug levels and oxidation parameters [144,146,147,148,154,156,157], and they do not verify the treatment compliance or the further impact in oxidative status. Furthermore, the distribution of unknown amounts of antioxidants in the usual diet intake could influence the results. As an example, the group of Roig-Revert et al. did check the initial adherence of all the participants to the Mediterranean diet and took this factor into account for statistical analysis [151].

Data concerning drug bioavailability were only reported by two research groups. Lafuente et al. analysed the erythrocyte content of DHA and obtained significant differences among supplemented and placebo groups [129,149]. Sanz-González et al. measured vitamin C levels and encountered inappropriate bioavailabilities, depending on different genetic expressions of SLC23A2 (which regulates intracellular levels of the vitamin), in the context of chronic hyperglycaemia and DR [145]. In addition, Haritoglou et al., who reported nonsignificant clinical results and did not measure supplemented α-lipoic acid levels, postulated that administered doses might have not been enough to produce retinal changes, because the ideal dose in humans has not yet been established in comparison to well-defined safety levels in animal assays [154].

In this context, bioavailability, the fraction of bioactive compound that reaches the blood circulation, is usually very low in nutraceuticals. In recent years, nanoformulations have been used in order to increase the bioavailability of phenolic compounds, vitamins and minerals [159]. Likewise, other nutraceuticals with more bioavailability have appeared, such as pterostilbene, a resveratrol analogue whose bioavailability in rats is 80% compared to 20% for resveratrol [160].

### 8.4. Conclusions

According to the results gleaned from the studies, it can be deduced that clinical changes were only observed after the longest follow-up periods, in terms of delaying the onset or reducing the progression of DR in T2DM. No other clinical effects could be observed, which means that antioxidant supplementation has not yet been proven to have an impact on BCVA or DME regression in the mid-term.

On the other hand, antioxidants did show an early influence on retinal function parameters after short follow-up periods, ranging from six to fourteen months. The profile of these patients was T1 or T2DM subjects presenting mild-to-moderate NPDR without CSME, or with DME but without the thickening of the retina in the OCT. These findings suggest that antioxidants are a valid prophylactic adjuvant therapy in the early stages of DR, in which anatomical damage is not excessive and there is no thickening of the central macula.

Taking into account the heterogeneity of the variables studied and the large diversity of antioxidants administered in the RCTs, we would recommend antioxidant oral supplementation with a IIb level of evidence in adult T1DM and T2DM patients without retinopathy or mild-to-moderate NPDR without DME.

In order to establish protocolled recommendations, larger sample sizes and longer follow-up periods should be accomplished in future clinical trials to determine the best antioxidant and the profile of candidates who will benefit from the adjuvant oral therapy in the mid- and long-term.

## 9. The Future

Despite the fact that the prevalence of diabetes is increasing worldwide, the advances in retinal imaging and new treatments have succeeded in reducing the rates of PDR and severe vision loss in developed countries.

The greater use of ultrawide-field photography of the fundus, which allows the evaluation of more than 80% of the retinal surface from a single image [161,162], and the improvement of noninvasive techniques, such as OCT angiography, for the early detection of microvascular damage in DR [53,163,164] will encourage better management of these patients, giving telemedicine a fundamental role in the next few years.

These advances, together with AI algorithms, to detect DR may predict the risk of retinopathy progression more efficiently and in early stages [165,166].

However, more than new diagnosis strategies, improved therapeutic approaches are still needed and are currently under investigation for the treatment of DR. These include emerging therapies for retinopathy that target alternative pathways for increased therapeutic effectiveness; alternative noninvasive delivery mechanisms or mechanisms providing a longer duration of action; approaches to prevent the onset of DR or to slow down the worsening of DR, such as oral antioxidant supplementation. In this review, we have staged the profile of patients who would benefit from antioxidant supplementation, adult T1DM and T2DM patients without retinopathy or mild-to-moderate NPDR without DME. However, new antioxidants with a higher bioavailability and better studies are needed to improve the level of evidence of this recommendation.

Finally, preclinical research suggests that gene therapy could be a promising therapeutic strategy for the future, eliminating the need for the frequent administration of anti-VEGF and increasing its brief therapeutic effect, which currently hinders the clinical practice [167].

## Figures and Tables

**Table 1:** International Clinical Disease Severity Scale for diabetic retinopathy (DR).

**Disease**	**Ophthalmoscopy Findings**
No apparent DR	No ocular findings
NPDR	No NV
Mild	Microaneurysms only
Moderate	Microaneurysms + blot haemorrhages, hard exudates, cotton wool spots (but less than in severe NPDR)
Severe	Intraretinal haemorrhages (≥20 in each quadrant) Definite venous beading (in two quadrants) Intraretinal microvascular abnormalities (IRMA) (in one quadrant) No signs of proliferative retinopathy
PDR	Neovascularisation or vitreous/preretinal haemorrhage/tractional retinal detachment
**DME = retinal thickening of hard exudates in the posterior pole**	
Mild	Some retinal thickening or hard exudates in the posterior pole but distant from the centre of the macula
Moderate	Retinal thickening or hard exudates approaching the centre of the macula but not involving the fovea
Severe	Retinal thickening or hard exudates involving the centre of the macula

DR = diabetic retinopathy; NPDR = nonproliferative diabetic retinopathy; NV = neovascularisation; PDR = proliferative diabetic retinopathy; DME = diabetic macular oedema.

**Table 2:** Management of DR patients depending on the pathology grade.

	No DR	NPDR			PDR	DME	
		Mild	Moderate	Severe		Non-Centre Involving	Centre
PDR progression risk within 1 year/3 years	5%/14%	12–27%/30.48%	52%/71%				
Referral to ophthalmologist	Not required	Not required	Required	Required	Required	Recommended if laser sources available *	Required
Treatment	Observation		Observation/PRP	PRP	Anti-VEGF/PRP/VPP	Laser: Focal/Grid	Anti-VEGF
Antioxidants role	Potentially indicated			Worthwhile?		Potentially indicated	
Follow-up	1–2 years	6–12 months/1–2 years *	3–6 months/6–12 months *	<3 months	<1 month If stabilised: 6 to 12 months	3 months	1 month

DR = diabetic retinopathy; NPDR = nonproliferative diabetic retinopathy; NV = neovascularisation; PDR = proliferative diabetic retinopathy; DME = diabetic macular oedema; PRP = panretinal photocoagulation; FA = fluorescein angiography; PPV = pars plana vitrectomy, VEGF = vascular endothelial growth factor. * Specific recommendations for low or intermediate resource settings [3].

**Table 3:** Studies of antioxidant supplementation in patients with diabetic retinopathy included in our review.

Author/Year/Country/Reference	Study	Study Focus	Antioxidant Composition per Pill	Trade Name Dose	N per Group: Supplemented (S) and Control (C). Mean Age (Years)	Follow-Up Time in Months	Clinical Findings	Biochemical Findings
Sanz-González 2020 Spain [145]	Case-control study	Type 2 DM with and without DR	Oil as a source of PUFAs: 400 mg Omega-3 (ω3): DHA 140 mg Vitamin C 80 mg Vitamin D 5 µg Vitamin B 20.1 mg Vitamin E 12 mg Lutein 6 mg Zeaxanthin 0.3 mg Glutathione 1 mg Hydroxytyrosol 0.75 mg Zinc 7.5 mg Copper 1 mg Selenium 55 µg Manganese 2 mg Dosage = 1 tablet/day: Supplement or Placebo	Nutrof Omega^®^ (Thea SA, (Barcelona, Spain)	N = 365 225 T2DM −With DR: 100 −Without DR: 125 140 healthy controls Mean Age: T2DM: 60 Controls: 55	38	The placebo group was more representative in subjects with T2DM in whom DR progressed. NS differences in IOP and CMT	The A/ω3 regime significantly reduced the pro-oxidants (*p* < 0.05) and augmented the antioxidants (*p* < 0.05).
Moon 2019 Korea [146]	Randomised (1:2:2), double-blind controlled trial	Type 2 DM with NPDR 40–80 y.o. AV > 0.5 Without laser or intravitreal therapy or intraocular surgery in the previous 6 months	S group 1: 50 mg—Grape seed proanthocyanidins extracts (GSPE) (Vitis vinifera extract) S group 2: 250 mg of calcium dobesilate (CD) C group.	GSPE: Entelon (Hanlim Pharm, Seoul, South Korea) CD: Doxium (Ilsung Pharm, Seoul, South Korea).	N = 86 3 tablets 3 times daily S1: GSPE (150 mg/day): 32 S2: CD (750 mg/day): 35 Placebo: 19	12	Hard exudates severity improvement: higher in GSPE (43.9%) vs. CD (14.29%) and vs. placebo (8%) (0.0007) NS differences between OCT parameters (CMT, TVM) GSPE TVM significantly decreases with respect to baseline.	NS differences with regard to vital signs and laboratory results between groups.
Lafuente 2019 Spain [129]	Randomised Single-Blind Controlled Trial	T2DM adults with decreased vision due to central-involved DME	Omega-3 Fatty Acids DHA 350 mg EPA 42.5 mg DPA 30 mg Vitamin C 26.7 mg Vitamin E 4 mg B vitamins 7.3 mg Lutein 3 mg Zeaxanthin 0.3 mg Glutathione 2 mg Zinc 1.66 mg Copper 0.16 mg Selenium 9.16 µg Manganese 0.33 mg	Brudyretina^®^ 1.5 g (Brudy Lab S.L Barcelona, Spain) 3 capsules of 1.5 g once daily	N = 55 (69 eyes) S + Ranibizumab * n = 26 (31 eyes) C: Only Ranibizumab * n = 29 (38 eyes) All patients with four monthly doses of ranibizumab followed by pro re nata basis.	36	VA: NS difference in ETDRS letters. Gains of >5 and >10 letters significantly higher in S group. CMT: Significant decrease in S group vs. C group (275 ±50 µm vs. 310 ± 97 µm) Number of Ranibizumab injections: NS differences between groups.	Significant differences in HbA1c, plasma total antioxidant capacity values, erythrocyte DHA content and IL-6 levels in favour of S group.
Sepahi 2018 Iran [147]	Phase 2 randomised, double-blind, placebo-controlled trial.	Refractory to conventional DME therapy in type 1 or 2 diabetes Refractory therapy including: macular photocoagulation and intravitreal injection of bevacizumab with or without triamcinolone	S1: Crocin tablet 15 mg S2: Crocin tablet 5 mg	Crocin tablet Pharmaceutical laboratory of School of Pharmacy, Mashhad University of Medical Science, Mashhad, Iran 1 tablet per day (15 mg, 5 mg or placebo)	N = 60 patients (101 eyes) S 1: 20 (33 eyes) S 2: 20 (34 eyes) C: 20 (34 eyes) Age: 41–82	Supplementation: 3 Follow-up: 6	VA: LogMAR: S1 significantly improved compared to S2 (*p* < 0.05) and to C (*p* = 0.02). CMT: S1 significantly improved compared to S2 (*p* < 0.05) and to C (*p* = 0.005). S2 NS improvement compared to C.	HbA1c and FBS: S1 and S2 significantly better than C.
Zhang 2017 China [148]	Randomised, double-blind, placebo-controlled trial	NPDR mild or moderate stages Type 2 diabetes Exclusion criteria: DME, other eye disorders other than mild or moderate NPDR	Lutein 10 mg Placebo capsule	Lutein 10 mg 1 capsule once a day (1 capsule of placebo once a day if C) Lutein Pharmaceutical Co Ltd. (Guangzhou, China)	N = 30 patients S: 15 C: 15 Mean age: 60.2., SD: 10.3	9	VA: slight NS improvement in S (*p* = 0.11) Contrast sensitivity: S: significant increase in 3 cycles/° by 0.16 (*p* = 0.02) ANOVA analysis showed differences between S and C. NS in 6.12 and 36 cycles/°. Glare sensitivity: NS differences.	
Lafuente 2017 Spain [149]	Randomised Single-Blind Controlled Trial	Type 2 diabetes adults with decreased vision due to central-involved DME.	Omega-3 Fatty Acids DHA 350 mg EPA 42.5 mg DPA 30 mg Vitamin C 26.7 mg Vitamin E 4 mg B vitamins 7.3 mg Lutein 3 mg Zeaxanthin 0.3 mg Glutathione 2 mg Zinc 1.66 mg Copper 0.16 mg Selenium 9.16 µg Manganese 0.33 mg	Brudyretina^®^ 1.5 g (Brudy Lab S.L Barcelona, Spain) 3 capsules of 1.5 g once daily	N = 76 eyes S + Ranibizumab * n = 34 C: Only Ranibizumab * n = 42 All patients with four monthly doses of ranibizumab followed by pro re nata basis.	24	VA: NS difference in ETDRS letters. Gains of >5 letters significantly higher in S group (*p* = 0.044), NS for gains of >10 letters. CMT: Significant decrease in S group (95% CI 7.20–97.656; *p* = 0.024) Number of Ranibizumab injections: NS differences between groups.	Significant increase in TAC (total antioxidative capacity) in S group (*p* < 0.001) Significant reduction in the erythrocyte membrane content of ω-6 arachidonic acid in the S group (*p* < 0.05) NS differences in HbA1c levels
Rodriguez-Carrizalez 2016 Mexico [150]	Randomised, controlled, phase IIa clinical trial	T2DM with NPDR, but without DME	S1: Ubiquinone 400 mg Dosage 1 tablet/day S2: Vitamin C 180 mg Vitamin E 30 mg Lutein 10 mg Astaxanthin 4 mg Zeaxanthin 1 mg Zinc 20 mg Dosage 1 tablet/day C: Placebo tablet	Noncommercialised supplement	N = 60 patients S1: N = 20 S2: N = 20 C: N = 20 Mean age S1: 58.5 ± 1.9 S2: 62.1 ±1.1 C: 57.8± 1.9	6	VA: NS changes	S1 and S2 Significant decrease in lipid peroxidation products, NO metabolites, catalase and glutathione peroxidase (*p* < 0.0001) Increased TAC (*p* < 0.0001) Vs. C group NS changes in HbA1c%, cholesterol and triglyceride levels between groups
Chous 2016 USA [144]	Randomised controlled clinical trial	T1 or T2DM without DR or with mild-to-moderate NPDR without CSME	S: Vitamin C 60 mg Vitamin D3 50 mg Vitamin E 40 mg α-Lipoic acid 150 mg Coenzyme Q10 20 mg Omega-3 Fatty Acids EPA 128 mg DHA 96 mg Zeaxanthin 8 mg Lutein 4 mg Zinc oxide 15 mg Benfotiamine N-acetyl cysteine Grape seed extract Resveratrol Turmeric root Extract green tea leaf Pycnogenol (Not specified mg) Dosage = 2 tablets/day C: Placebo tablet	DiVFuSS^®^ (ZeaVision, LLC, Chesterfield, MO, USA)	N = 67 patients S: N = 39 C: N = 28 Mean age S: 53.5 ± 14.6 C: 59.7 ± 10.3	6	VA: NS changes CMT: NS changes RNFL thickness: NS changes Contrast sensitivity, colour error Score, visual field mean sensitivity and MPOD: significant 27% improvement in the S group vs. 2% in the C group. (*p* values ranging from 0.008 to <0.0001). MPOD (macular pigment optical density)	NS changes in HbA1c, total cholesterol or TNF-α between the groups
Roig-Revert 2015 Spain [151]	Randomised, prospective, multicentre study	T2DM Group 1: NPDR ± DME Group 2: Diabetic patients without DR Healthy subjects	S: Vitamin C 80 mg Vitamin D 5 µg Vitamin B 20.1 mg Vitamin E 12 mg Omega-3: DHA 140 mg Lutein 6 mg Zeaxanthin 0.3 mg Glutathione 1 mg Hydroxytyrosol 0.75 mg Zinc 7.5 mg Copper 1 mg Selenium 55 µg Manganese 2 mg Dosage = 1 tablet/day C: no placebo capsule	Nutrof Omega^®^ (Thea SA, (Barcelona, Spain)	N = 208 patients Group 1 DM DR+ (N = 62) S (n = not specified) C (n = not specified) Group 2 DM DR- (N = 68) S (N = not specified) C (n = not specified) Group 3 Healthy subjects (N = 78) S (n = not specified) C (n = not specified) Mean age DM DR+ 65.1 ± 8.6 DM DR− 62.3 ± 10.1	18	Group 1 DM DR + DR progression: S: 61% C: 91% Group 2 DM DR- DR onset: S: 9% C: 35% RNFLT of the LE was significantly reduced in the S group (*p* = 0.01)	Significant reduction in TAS in supplemented DMDR+ (*p* = 0.020) Plasma lipid peroxidation by-products significantly decreased in the DMDR+ supplemented group. NS in terms of HbA1c, HDL/LDL cholesterol and triglycerides.
Domanico 2015 Italy [152]	Randomised prospective study	T2DM showing mild-to-moderate NPDR, without CSME or CVRF	Vitamin E 30 mg Pycnogenol 50 mg Coenzyme Q10 20 mg Dosage = 1 tablet/day C: no placebo capsule	Diaberet^®^ (Visufarma, Rome, Italy)	N = 68 patients (eyes) S: N = 34 C: N = 34 Mean age S: 58.29 ± 12.37 C: 62.29 ± 11.54	6	CMT: significant reduction on the S group (*p* < 0.01) (–15.44 µm, [95% CI: 3.26, 27.61])	Significant reduction of ROS levels (free oxygen radical test) in the S group (*p* < 0.001)
Watanabe 2014 Japan [153]	Randomised, prospective study	T2DM patients without DR	2.5 g of goshajinkigan extract three times a day, which included: 4.5 g of the compound extracts of 10 herbal medicines: Rehmanniae radix (5 g), Achyranthis radix (3 g), Corni fructus (3 g), Dioscoreae rhizoma (3 g), Hoelen (3 g), Plantaginis semen (3 g), Alismatis rhizoma (3 g), Moutan cortex (3 g), Cinnamomi cortex (1 g) and heat-processed Aconiti radix (1 g)	TJ-107; Tsumura Co., Tokyo, Japan	N = 116 patients S: N = 74 C: N = 42 Mean age S: 59.4 ± 7.8 C: 60.9 ± 7.4	60	Progression of retinopathy: No differences between S and C. A total of 25 patients had DR at the end of the study. 17.9% in Goshajinkigan group 20.0% in control group *p* = 0.816	Glycated haemoglobin significantly decreased in the S group at the 60th month. Fasting glucose significantly decreased in the S group beginning at the 36th month. No differences between insulin or oral antidiabetic medications.
Haritoglou 2011 Germany [154]	Randomised, prospective, multicentre, study	T2DM showing mild-to-moderate NPDR in at least one eye	S: α-lipoic acid (ALA) 600 mg Dosage 1 tablet/day C: placebo tablet	Noncommercialised supplement	N: = 399 patients S: = 196 C: = 203 Mean age S 58.0 C 57.9	24	CSME debut during follow-up S 26/196 C 30/203 NS reduction in macular oedema development (*p* = 0.7108)	NS differences in terms of HbA1c levels between groups
García-Medina 2011 Spain [155]	Randomised prospective study	T2DM with NPDR but no CSME	S: Vitamin C 60 mg Vitamin E 10 mg Lutein 3 mg Zinc 13.5 mg Copper 1 mg Selenium 10 µg Manganese 1 mg Niacin 10 mg β-Carotene 3 mg Dosage = 2 tablets/day C: no placebo capsule	Vitalux Forte^®^ (Novartis Pharma AG Ophthalmics, Basel, Switzerland)	N = 97 patients S: N = 56 C: N = 41 Mean age S 53.3 ± 11.9 C 57.0 ± 11.4	60	VA: NS changes DR degree: Significant progression in C group (*p* < 0.01) vs. non-significant progression in S group	Significant reduced plasma lipid peroxidation end products (MDA) in S vs. increased in C group (*p* < 0.01) Stable TAS in the S group vs. significant decrease in C group (*p* = 0.02)
Forte 2011 Italy [156]	Randomised prospective, interventional, controlled study	T2DM and DME without macular thickening at OCT	S = Desmin 300 mg Troxerutin 300 mg C. asiatica 30 mg Melilotus 160 Dosage 1/day C = Placebo capsule	Noncommercialised supplement	N = 40 patients (eyes) S = 20 C = 20 Mean age S 63.6 ± 3.1 C 62.2 ± 3.4.	14	VA: NS differences CMT: NS differences between groups. Five eyes of the S group showed resolution of retinal cysts, in comparison to no changes in the C group RS (dB): S showed a significant increase at month 14 (*p* < 0.001) (16.43 ± 0.39)	NS differences during follow-up in terms of HbA1c, microalbuminuria or blood pressure
Bursell 1999 USA [157]	Randomised double-masked placebo-controlled crossover trial	T1DM without or with minimal DR	S = Vitamin E 1800 IU C = Placebo capsule Dosage 1800 IU/day	Noncommercialised supplement	N = 45 patients S = 36 (T1DM) C = 9 (ND) 4 months follow-up Crossover S = 9 (ND) C = 36 (T1DM) 4 months follow-up Mean age DM = 31.2 ± 6.8 ND = 31.6 ± 7.1	8	T1DM significant increase in retinal blood flow (*p* < 0.001) (34.5 ± 7.8 pixel^2^/s) Retinal blood flow measured by mean circulation times in fluorescein angiography: C: No changes	NS differences in terms of HbA1c between groups Statistically significant creatinine clearance improvement after supplementation in T1DM subjects (*p* = 0.039). This change reverted after crossover.

C = Control group, CMT = Central Macular Thickness, CSME = Clinically significant macular oedema, DHA = Docosahexaenoic acid, DM = Diabetes Mellitus, DM DR + = Diabetic patients with diabetic retinopathy, DM DR = Diabetic patients without diabetic retinopathy, DME = diabetic macular oedema, DPA = Docosapentaenoic acid, DR = Diabetic Retinopathy, EPA = Eicosapentaenoic acid, ETDRS = Early Treatment Diabetic Retinopathy Study Scale, FBS = fasting blood sugar, HbA1C = glycated haemoglobin, IOP = Intraocular pressure, IU = International Units, MDA = Malondialdehyde, MPOD = macular pigment optical density, NO = nitrogen oxide. NPDR = Nonproliferative diabetic retinopathy, NS = Not statistically significant, PUFA = polyunsaturated fatty acids, RS = retinal sensitivity, S = Supplemented group, T2DM = Type 2 Diabetes Mellitus, TAS = Total Antioxidant Status, TVM = total macular volume, VA = visual acuity. * Ranibizumab dosage: four loading doses followed by pro re nata treatment, both groups.

## Supplementary Materials

The following are available online at https://www.mdpi.com/article/10.3390/ijms22084020/s1, File S1: Inclusion and exclusion criteria, searches and excluded studies in the systematic review of clinical studies evaluating the effect of antioxidant supplementation in diabetic retinopathy (DR).
