# Supplementary material for: Role of Oral Antioxidant Supplementation in the Current Management of Diabetic Retinopathy
Source: Int J Mol Sci. 2021 Apr 13;22(8):4020. doi: 10.3390/ijms22084020 (PMC8069935; doi:10.3390/ijms22084020)
Supplement: Supplementary file 1 [file ijms-22-04020-s001.pdf]

Appendix A

Inclusion and exclusion criteria, searches and excluded studies in the systematic review of clinical studies evaluating the effect of antioxidant supplementation in diabetic retinopathy (DR).

Inclusion criteria

1. Human experimental studies about antioxidant supplementation.
2. Patients with DR or analysis of DR development.
3. Follow-up time of 6 or more months
4. People included at the study: 30 or more
5. Articles in English, Spanish or French

Exclusion criteria

1. Not an experimental study about antioxidant supplementation in humans.
2. No DR patients included.
3. Follow-up time under 6 months
4. Less than 30 people included.
5. Not in English, Spanish or French

Searches:

A systematic review in Pubmed (NLM) database was conducted through EndNote^{x9} (Claritative Analytics, Chandler, Az, USA) program.

1. “Antioxidants” (abstract) AND “diabetic retinopathy” (abstract) AND “trial” (abstract)
    a. 29 results
2. “Supplementation” (abstract) AND “diabetic retinopathy” (abstract) AND “trial” (abstract)
    a. 27 results
3. “Oral supplement” (abstract) AND “retinopathy” (abstract)
    a. 14 results
4. “Antioxidant supplement” (all fields) AND “diabetic retinopathy” (all fields)
    a. 50 results
5. “Extract” (abstract) AND “diabetic retinopathy” (abstract) AND “trial” (abstract)
    a. 5 results

Results, studies included (bold) and excluded with the cause of exclusion:

<u>1. “Antioxidants” (abstract) AND “diabetic retinopathy” (abstract) AND “trial” (abstract):</u>

1. Garcia-Medina JJ, Rubio-Velazquez E, Foulquie-Moreno E, Casaroli-Marano RP, Pinazo-Duran MD, Zanon-Moreno V, et al. Update on the Effects of Antioxidants on Diabetic Retinopathy: In Vitro Experiments, Animal Studies and Clinical Trials. Antioxidants (Basel). 2020;9, 10.3390/antiox9060561

1. Review

2. Lafuente M, Ortin L, Argente M, Guindo JL, Lopez-Bernal MD, Lopez-Roman FJ, et al. Three-Year Outcomes in a Randomized Single-Blind Controlled Trial of Intravitreal Ranibizumab and Oral Supplementation with

Docosahexaenoic Acid and Antioxidants for Diabetic Macular Edema. Retina. 2019;39, 1083-90. 10.1097/IAE.0000000000002114´

Included

3. She C, Shang F, Zhou K, Liu N. Serum Carotenoids and Risks of Diabetes and Diabetic Retinopathy in a Chinese Population Sample. Curr Mol Med. 2017;17, 287-97. 10.2174/1566524017666171106112131

1. Cross-sectional population study

4. Lafuente M, Ortin L, Argente M, Guindo JL, Lopez-Bernal MD, Lopez-Roman FJ, et al. COMBINED INTRAVITREAL RANIBIZUMAB AND ORAL SUPPLEMENTATION WITH DOCOSAHEXAENOIC ACID AND ANTIOXIDANTS FOR DIABETIC MACULAR EDEMA: Two-Year Randomized Single-Blind Controlled Trial Results. Retina. 2017;37, 1277-86. 10.1097/IAE.0000000000001363

Included

5. Rodriguez-Carrizalez AD, Castellanos-Gonzalez JA, Martinez-Romero EC, Miller-Arrevillaga G, Pacheco-Moises FP, Roman-Pintos LM, et al. The effect of ubiquinone and combined antioxidant therapy on oxidative stress markers in non-proliferative diabetic retinopathy: A phase IIa, randomized, double-blind, and placebo-controlled study. Redox Rep. 2016;21, 155-63. 10.1179/1351000215Y.0000000040

Included

6. Kowluru RA, Mishra M. Oxidative stress, mitochondrial damage and diabetic retinopathy. Biochim Biophys Acta. 2015;1852, 2474-83. 10.1016/j.bbadis.2015.08.001

1. Revisión

7. Rodriguez-Carrizalez AD, Castellanos-Gonzalez JA, Martinez-Romero EC, Miller-Arrevillaga G, Roman-Pintos LM, Pacheco-Moises FP, et al. The antioxidant effect of ubiquinone and combined therapy on mitochondrial function in blood cells in non-proliferative diabetic retinopathy: A randomized, double-blind, phase IIa, placebo-controlled study. Redox Rep. 2016;21, 190-5. 10.1179/1351000215Y.0000000032

Included

8. Gramajo AL, Marquez GE, Torres VE, Juarez CP, Rosenstein RE, Luna JD, et al. Therapeutic benefit of melatonin in refractory central serous chorioretinopathy. Eye (Lond). 2015;29, 1036-45. 10.1038/eye.2015.104

2. Central serous chorioretinopathy

9. Chous AP, Richer SP, Gerson JD, Kowluru RA. The Diabetes Visual Function Supplement Study (DiVFuSS). Br J Ophthalmol. 2016;100, 227-34. 10.1136/bjophthalmol-2014-306534

Included

10. Domanico D, Fragiotta S, Cutini A, Carnevale C, Zompatori L, Vingolo EM. Circulating levels of reactive oxygen species in patients with nonproliferative diabetic retinopathy and the influence of antioxidant supplementation: 6-month follow-up. Indian J Ophthalmol. 2015;63, 9-14. 10.4103/0301-4738.151455

Included

11. Dutta S, Islam MN, Chakroborty S, Mondal A, Bandopadhay R, Gayen S, et al. Effect of anti-oxidant on tear film in patients suffering from diabetes mellitus. J Indian Med Assoc. 2014;112, 108-9.

3. 30 days

12. Tanaka S, Yoshimura Y, Kawasaki R, Kamada C, Tanaka S, Horikawa C, et al. Fruit intake and incident diabetic retinopathy with type 2 diabetes. Epidemiology. 2013;24, 204-11. 10.1097/EDE.0b013e318281725e

1.Not an antioxidant clinical trial.

13. Chan KH, O'Connell RL, Sullivan DR, Hoffmann LS, Rajamani K, Whiting M, et al. Plasma total bilirubin levels predict amputation events in type 2 diabetes mellitus: the Fenofibrate Intervention and Event Lowering in Diabetes (FIELD) study. Diabetologia. 2013;56, 724-36. 10.1007/s00125-012-2818-4

1. Not an antioxidant clinical trial.

14. Berson EL, Rosner B, Sandberg MA, Weigel-DiFranco C, Willett WC. omega-3 intake and visual acuity in patients with retinitis pigmentosa receiving vitamin A. Arch Ophthalmol. 2012;130, 707-11. 10.1001/archophthalmol.2011.2580

2. Retinitis pigmentosa

15. Kowluru RA, Zhong Q. Beyond AREDS: is there a place for antioxidant therapy in the prevention/treatment of eye disease? Invest Ophthalmol Vis Sci. 2011;52, 8665-71. 10.1167/iovs.10-6768

1. Review

16. Nebbioso M, Federici M, Rusciano D, Evangelista M, Pescosolido N. Oxidative stress in preretinopathic diabetes subjects and antioxidants. Diabetes Technol Ther. 2012;14, 257-63. 10.1089/dia.2011.0172

3. 30 days

17. Haritoglou C, Gerss J, Hammes HP, Kampik A, Ulbig MW, Group RS. Alpha-lipoic acid for the prevention of diabetic macular edema. Ophthalmologica. 2011;226, 127-37. 10.1159/000329470

Included

18. Garcia-Medina JJ, Pinazo-Duran MD, Garcia-Medina M, Zanon-Moreno V, Pons-Vazquez S. A 5-year follow-up of antioxidant supplementation in type 2 diabetic retinopathy. Eur J Ophthalmol. 2011;21, 637-43. 10.5301/EJO.2010.6212

Included

19. Babizhayev MA, Micans P, Guiotto A, Kasus-Jacobi A. N-acetylcarnosine lubricant eyedrops possess all-in-one universal antioxidant protective effects of L-carnosine in aqueous and lipid membrane environments, aldehyde scavenging, and transglycation activities inherent to cataracts: a clinical study of the new vision-saving drug N-acetylcarnosine eyedrop therapy in a database population of over 50,500 patients. Am J Ther. 2009;16, 517-33. 10.1097/MJT.0b013e318195e327

1. No clinical trial

20. Babizhayev MA, Kasus-Jacobi A. State of the art clinical efficacy and safety evaluation of N-acetylcarnosine dipeptide ophthalmic prodrug. Principles for the delivery, self-bioactivation, molecular targets and interaction with a highly evolved histidyl-hydrazide structure in the treatment and therapeutic management of a group of sight-threatening eye diseases. Curr Clin Pharmacol. 2009;4, 4-37. 10.2174/157488409787236074

1. No clinical trial

2. Cataracts

21. Chandra P, Hegde KR, Varma SD. Possibility of topical antioxidant treatment of cataracts: corneal penetration of pyruvate in humans. Ophthalmologica. 2009;223, 136-8. 10.1159/000184538

2. Cataract

22. Cangemi FE. TOZAL Study: an open case control study of an oral antioxidant and omega-3 supplement for dry AMD. BMC Ophthalmol. 2007;7, 3. 10.1186/1471-2415-7-3

2. Age-related macular disease (AMD)

23. Schmidt-Erfurth U. Nutrition and retina. Dev Ophthalmol. 2005;38, 120-47. 10.1159/000082772

1. Review

24. Bandello F, Pognuz R, Polito A, Pirracchio A, Menchini F, Ambesi M. Diabetic macular edema: classification, medical and laser therapy. Semin Ophthalmol. 2003;18, 251-8. 10.1080/08820530390895262

1. Review

25. Chiarelli F, Santilli F, Sabatino G, Blasetti A, Tumini S, Cipollone F, et al. Effects of vitamin E supplementation on intracellular antioxidant enzyme production in adolescents with type 1 diabetes and early microangiopathy. Pediatr Res. 2004;56, 720-5. 10.1203/01.PDR.0000141990.12375.13

4. Less than 30 study subjects

26. Inukai T, Takanashi K, Tayama K, Aso Y, Takemura Y. High glucose concentrations abolish the superoxide dismutase response of leukocytes to ascorbic acid or troglitazone in type 2 diabetes mellitus. Life Sci. 2002;70, 2391-401. 10.1016/s0024-3205(02)01513-8

1. Not an antioxidant clinical trial.

27. Polunin GS, Makarov IA, Shirshikov Iu K, Makashova NV. [The efficacy of the antioxidant preparation Histochrome in the treatment of hemophthalmos in hypertension and diabetes mellitus]. Vestn Oftalmol. 2000;116, 19-20.

5. Not in English, Spanish, french

28. Fujita H, Narita T, Meguro H, Shimotomai T, Kitazato H, Kagaya E, et al. No association of glutathione S-transferase M1 gene polymorphism with diabetic nephropathy in Japanese type 2 diabetic patients. Ren Fail. 2000;22, 479-86. 10.1081/jdi-100100889

1. Not an antioxidant clinical trial.

29. Kahler W, Kuklinski B, Ruhlmann C, Plotz C. [Diabetes mellitus--a free radical-associated disease. Results of adjuvant an

3. Less than 6 months

<u>2: “Supplementation” (abstract) AND “diabetic retinopathy” (abstract) AND “trial” (abstract)</u>

1. Tecilazich F, Formenti AM, Giustina A. Role of vitamin D in diabetic retinopathy: Pathophysiological and clinical aspects. Rev Endocr Metab Disord. 202010.1007/s11154-020-09575-4

1. Review

2. Schwartz SG, Wang X, Chavis P, Kuriyan AE, Abariga SA. Vitamin A and fish oils for preventing the progression of retinitis pigmentosa. Cochrane Database Syst Rev. 2020;6, CD008428. 10.1002/14651858.CD008428.pub3

2. Retinitis pigmentosa

3. Omidian M, Mahmoudi M, Javanbakht MH, Eshraghian MR, Abshirini M, Daneshzad E, et al. Effects of vitamin D supplementation on circulatory YKL-40 and MCP-1 biomarkers associated with vascular diabetic complications: A randomized, placebo-controlled, double-blind clinical trial. Diabetes Metab Syndr. 2019;13, 2873-7. 10.1016/j.dsx.2019.07.047

3. 12 weeks

4. Richter J, Zavorkova M, Vetvicka V, Liehneova I, Kral V, Rajnohova Dobiasova L. Effects of beta-glucan and Vitamin D Supplementation on Inflammatory Parameters in Patients with Diabetic Retinopathy. J Diet Suppl. 2019;16, 369-78. 10.1080/19390211.2018.1458769

3. 3 months

5. Lafuente M, Ortin L, Argente M, Guindo JL, Lopez-Bernal MD, Lopez-Roman FJ, et al. Three-Year Outcomes in a Randomized Single-Blind Controlled Trial of Intravitreal Ranibizumab and Oral Supplementation with Docosahexaenoic Acid and Antioxidants for Diabetic Macular Edema. Retina. 2019;39, 1083-90. 10.1097/IAE.0000000000002114

Included

6. Kheirouri S, Naghizadeh S, Alizadeh M. Zinc supplementation does not influence serum levels of VEGF, BDNF, and NGF in diabetic retinopathy patients: a randomized controlled clinical trial. Nutr Neurosci. 2019;22, 718-24. 10.1080/1028415X.2018.1436236

3. 3 months

7. Zhang PC, Wu CR, Wang ZL, Wang LY, Han Y, Sun SL, et al. Effect of lutein supplementation on visual function in nonproliferative diabetic retinopathy. Asia Pac J Clin Nutr. 2017;26, 406-11. 10.6133/apjcn.032016.13

Included

8. Lafuente M, Ortin L, Argente M, Guindo JL, Lopez-Bernal MD, Lopez-Roman FJ, et al. COMBINED INTRAVITREAL RANIBIZUMAB AND ORAL SUPPLEMENTATION WITH DOCOSAHEXAENOIC ACID AND ANTIOXIDANTS FOR DIABETIC MACULAR EDEMA: Two-Year Randomized Single-Blind Controlled Trial Results. Retina. 2017;37, 1277-86. 10.1097/IAE.0000000000001363

Included

9. Gong X, Rubin LP. Role of macular xanthophylls in prevention of common neovascular retinopathies: retinopathy of prematurity and diabetic retinopathy. Arch Biochem Biophys. 2015;572, 40-8. 10.1016/j.abb.2015.02.004

1. Review

10. Domanico D, Fragiotta S, Cutini A, Carnevale C, Zompatori L, Vingolo EM. Circulating levels of reactive oxygen species in patients with nonproliferative diabetic retinopathy and the influence of antioxidant supplementation: 6-month follow-up. Indian J Ophthalmol. 2015;63, 9-14. 10.4103/0301-4738.151455

Included

11. Gerstenblith AT, Baskin DE, Shah CP, Wolfe JD, Fineman MS, Kaiser RS, et al. Electroretinographic effects of omega-3 Fatty Acid supplementation on dry age-related macular degeneration. JAMA Ophthalmol. 2013;131, 365-9. 10.1001/jamaophthalmol.2013.642

2. AMD

12. Weigert G, Kaya S, Pemp B, Sacu S, Lasta M, Werkmeister RM, et al. Effects of lutein supplementation on macular pigment optical density and visual acuity in patients with age-related macular degeneration. Invest Ophthalmol Vis Sci. 2011;52, 8174-8. 10.1167/iovs.11-7522

2. AMD

13. Garcia-Medina JJ, Pinazo-Duran MD, Garcia-Medina M, Zanon-Moreno V, Pons-Vazquez S. A 5-year follow-up of antioxidant supplementation in type 2 diabetic retinopathy. Eur J Ophthalmol. 2011;21, 637-43. 10.5301/EJO.2010.6212

Included

14. Lee TK, Clandinin MT, Hebert M, MacDonald IM. Effect of docosahexaenoic acid supplementation on the macular function of patients with Best vitelliform macular dystrophy: randomized clinical trial. Can J Ophthalmol. 2010;45, 514-9. 10.3129/i10-028

2. Best vitelliform macular dystrophy

15. Berson EL, Rosner B, Sandberg MA, Weigel-DiFranco C, Brockhurst RJ, Hayes KC, et al. Clinical trial of lutein in patients with retinitis pigmentosa receiving vitamin A. Arch Ophthalmol. 2010;128, 403-11. 10.1001/archophthalmol.2010.32

Included

16. Altaweel MM, Hanzlik RP, Ver Hoeve JN, Eells J, Zhang B. Ocular and systemic safety evaluation of calcium formate as a dietary supplement. J Ocul Pharmacol Ther. 2009;25, 223-30. 10.1089/jop.2008.0128

1. Security study

17. Zeng K, Xu H, Mi M, Zhang Q, Zhang Y, Chen K, et al. Dietary taurine supplementation prevents glial alterations in retina of diabetic rats. Neurochem Res. 2009;34, 244-54. 10.1007/s11064-008-9763-0

1. Animal trial

18. Cangemi FE. TOZAL Study: an open case control study of an oral antioxidant and omega-3 supplement for dry AMD. BMC Ophthalmol. 2007;7, 3. 10.1186/1471-2415-7-3

1. AMD

19. Schmidt-Erfurth U. Nutrition and retina. Dev Ophthalmol. 2005;38, 120-47. 10.1159/000082772

1. Review

20. Chiarelli F, Santilli F, Sabatino G, Blasetti A, Tumini S, Cipollone F, et al. Effects of vitamin E supplementation on intracellular antioxidant enzyme production in adolescents with type 1 diabetes and early microangiopathy. Pediatr Res. 2004;56, 720-5. 10.1203/01.PDR.0000141990.12375.13

4. Less than 30 subjects

21. Bhartiya P, Sharma P, Biswas NR, Tandon R, Khokhar SK. Levodopa-carbidopa with occlusion in older children with amblyopia. J AAPOS. 2002;6, 368-72. 10.1067/mpa.2002.129043

2. Amblyopia

22. Bursell SE, Clermont AC, Aiello LP, Aiello LM, Schlossman DK, Feener EP, et al. High-dose vitamin E supplementation normalizes retinal blood flow and creatinine clearance in patients with type 1 diabetes. Diabetes Care. 1999;22, 1245-51. 10.2337/diacare.22.8.1245
Included

23. van der Pijl JW, van der Woude FJ, Swart W, van Es LA, Lemkes HH. Effect of danaparoid sodium on hard exudates in diabetic retinopathy. Lancet. 1997;350, 1743-5. 10.1016/S0140-6736(97)07126-2

3. 6 weeks

4. 9 patients

24. Faure P, Benhamou PY, Perard A, Halimi S, Roussel AM. Lipid peroxidation in insulin-dependent diabetic patients with early retina degenerative lesions: effects of an oral zinc supplementation. Eur J Clin Nutr. 1995;49, 282-8.

4. 22 patients

25. Kahler W, Kuklinski B, Ruhlmann C, Plotz C. [Diabetes mellitus--a free radical-associated disease. Results of adjuvant antioxidant supplementation]. Z Gesamte Inn Med. 1993;48, 223-32.

3. 3 months

26. Mori TA, Vandongen R, Masarei JR. Fish oil-induced changes in apolipoproteins in IDDM subjects. Diabetes Care. 1990;13, 725-32. 10.2337/diacare.13.7.725

4. 22 patients

27. Clements RS, Jr. New therapies for the chronic complications of older diabetic patients. Am J Med. 1986;80, 54-60. 10.1016/0002-9343(86)90537-1

1. Review

3: oral supplement (abstract) & retinopathy (abstract)

1. Mu PW, Tang XX, Tan Y, Wang YN, Lin S, Wang MM, et al. Effect of basal insulin supplement therapy on diabetic retinopathy in short-duration type 2 diabetes: A one-year randomized parallel-group trial. J Diabetes. 2019;11, 949-57. 10.1111/1753-0407.12928

1. Not an antioxidant clinical trial.

2. Khalesi N, Bordbar A, Khosravi N, Kabirian M, Karimi A. The Efficacy of Omega-3 Supplement on Prevention of Retinopathy of Prematurity in Premature Infants: A Randomized Double-blinded Controlled trial. Curr Pharm Des. 2018;24, 1845-8. 10.2174/1381612824666180601094849

2. Retinopathy of prematurity

3. Sepahi S, Mohajeri SA, Hosseini SM, Khodaverdi E, Shoeibi N, Namdari M, et al. Effects of Crocin on Diabetic Maculopathy: A Placebo-Controlled Randomized Clinical Trial. Am J Ophthalmol. 2018;190, 89-98. 10.1016/j.ajo.2018.03.007

Included

4. Lafuente M, Ortin L, Argente M, Guindo JL, Lopez-Bernal MD, Lopez-Roman FJ, et al. Three-Year Outcomes in a Randomized Single-Blind Controlled Trial of Intravitreal Ranibizumab and Oral Supplementation with Docosahexaenoic Acid and Antioxidants for Diabetic Macular Edema. Retina. 2019;39, 1083-90. 10.1097/IAE.0000000000002114

Included

5. Kim J, Jo K, Kim CS, Kim JS. Aster koraiensis extract prevents diabetes-induced retinal vascular dysfunction in spontaneously diabetic Torii rats. BMC Complement Altern Med. 2017;17, 497. 10.1186/s12906-017-1998-3

1. Animal study

6. Lafuente M, Ortin L, Argente M, Guindo JL, Lopez-Bernal MD, Lopez-Roman FJ, et al. COMBINED INTRAVITREAL RANIBIZUMAB AND ORAL SUPPLEMENTATION WITH DOCOSAHEXAENOIC ACID AND ANTIOXIDANTS FOR DIABETIC MACULAR EDEMA: Two-Year Randomized Single-Blind Controlled Trial Results. Retina. 2017;37, 1277-86. 10.1097/IAE.0000000000001363

Included

7. Domanico D, Fragiotta S, Cutini A, Carnevale C, Zompatori L, Vingolo EM. Circulating levels of reactive oxygen species in patients with nonproliferative diabetic retinopathy and the influence of antioxidant supplementation: 6-month follow-up. Indian J Ophthalmol. 2015;63, 9-14. 10.4103/0301-4738.151455

Included

8. Ghadiri Soufi F, Arbabi-Aval E, Rezaei Kanavi M, Ahmadieh H. Anti-inflammatory properties of resveratrol in the retinas of type 2 diabetic rats. Clin Exp Pharmacol Physiol. 2015;42, 63-8. 10.1111/1440-1681.12326

1. Animal study

9. Howard KP, Klein BE, Dreyer JO, Danforth LG, Klein R. Cross-sectional associations of medication and supplement use with retinal vascular diameter in the Beaver Dam Eye Study. JAMA Ophthalmol. 2014;132, 23-31. 10.1001/jamaophthalmol.2013.6326

1. Cross-sectional study

10. Soufi FG, Mohammad-Nejad D, Ahmadieh H. Resveratrol improves diabetic retinopathy possibly through oxidative stress - nuclear factor kappaB - apoptosis pathway. Pharmacol Rep. 2012;64, 1505-14. 10.1016/s1734-1140(12)70948-9

1. Rats

11. Richer SP, Stiles W, Graham-Hoffman K, Levin M, Ruskin D, Wrobel J, et al. Randomized, double-blind, placebo-controlled study of zeaxanthin and visual function in patients with atrophic age-related macular degeneration: the Zeaxanthin and Visual Function Study (ZVF) FDA IND #78, 973. Optometry. 2011;82, 667-80 e6. 10.1016/j.optm.2011.08.008

2. AMD

12. Lee TK, Clandinin MT, Hebert M, MacDonald IM. Effect of docosahexaenoic acid supplementation on the macular function of patients with Best vitelliform macular dystrophy: randomized clinical trial. Can J Ophthalmol. 2010;45, 514-9. 10.3129/i10-028

2. Best vitelliform macular dystrophy

13. Altaweel MM, Hanzlik RP, Ver Hoeve JN, Eells J, Zhang B. Ocular and systemic safety evaluation of calcium formate as a dietary supplement. J Ocul Pharmacol Ther. 2009;25, 223-30. 10.1089/jop.2008.0128

1. Safety study

14. Cangemi FE. TOZAL Study: an open case control study of an oral antioxidant and omega-3 supplement for dry AMD. BMC Ophthalmol. 2007;7, 3. 10.1186/1471-2415-7-3

2. AMD

4: “Antioxidant supplement” (all fields) and “Diabetic retinopathy” (all fields)

1. Zhang Y, Gao Z, Gao X, Yuan Z, Ma T, Li G, et al. Tilianin Protects Diabetic Retina through the Modulation of Nrf2/TXNIP/NLRP3 Inflammasome Pathways. J Environ Pathol Toxicol Oncol. 2020;39, 89-99. 10.1615/JEnvironPatholToxicolOncol.2020032544

1. Animal study

2. She C, Shang F, Cui M, Yang X, Liu N. Association between dietary antioxidants and risk for diabetic retinopathy in a Chinese population. Eye (Lond). 202010.1038/s41433-020-01208-z

1. Not an antioxidant clinical trial.

3. Sanz-Gonzalez SM, Garcia-Medina JJ, Zanon-Moreno V, Lopez-Galvez MI, Galarreta-Mira D, Duarte L, et al. Clinical and Molecular-Genetic Insights into the Role of Oxidative Stress in Diabetic Retinopathy: Antioxidant Strategies and Future Avenues. Antioxidants (Basel). 2020;9, 10.3390/antiox9111101

Included

4. Saenz de Viteri M, Hernandez M, Bilbao-Malave V, Fernandez-Robredo P, Gonzalez-Zamora J, Garcia-Garcia L, et al. A Higher Proportion of Eicosapentaenoic Acid (EPA) When Combined with Docosahexaenoic Acid (DHA) in Omega-3 Dietary Supplements Provides Higher Antioxidant Effects in Human Retinal Cells. Antioxidants (Basel). 2020;9, 10.3390/antiox9090828

1. In vitro study

5. Li LH, Lee JC, Leung HH, Lam WC, Fu Z, Lo ACY. Lutein Supplementation for Eye Diseases. Nutrients. 2020;12, 10.3390/nu12061721

1. Review

6. Lai TT, Yang CM, Yang CH. Astaxanthin Protects Retinal Photoreceptor Cells against High Glucose-Induced Oxidative Stress by Induction of Antioxidant Enzymes via the PI3K/Akt/Nrf2 Pathway. Antioxidants (Basel). 2020;9, 10.3390/antiox9080729

1. Animal study

7. Kim YS, Kim J, Kim CS, Lee IS, Jo K, Jung DH, et al. The Herbal Combination CPA4-1 Inhibits Changes in Retinal Capillaries and Reduction of Retinal Occludin in db/db Mice. Antioxidants (Basel). 2020;9, 10.3390/antiox9070627

1. Animal study

8. Battaglia Parodi M, Brunoro A, Tomasso L, Scuderi G. Benefits of micronutrient supplementation for reducing the risk of wet age-related macular disease and diabetic retinopathy: An update. Eur J Ophthalmol. 2020;30, 780-94. 10.1177/1120672120920537

1. Review

9. Tabatabaei-Malazy O, Ardeshirlarijani E, Namazi N, Nikfar S, Jalili RB, Larijani B. Dietary antioxidative supplements and diabetic retinopathy; a systematic review. J Diabetes Metab Disord. 2019;18, 705-16. 10.1007/s40200-019-00434-x

1. Review

10. Rossino MG, Casini G. Nutraceuticals for the Treatment of Diabetic Retinopathy. Nutrients. 2019;11, 10.3390/nu11040771´

1. Review

11. Ren YB, Qi YX, Su XJ, Luan HQ, Sun Q. Therapeutic effect of lutein supplement on non-proliferative diabetic retinopathy: A retrospective study. Medicine (Baltimore). 2019;98, e15404. 10.1097/MD.0000000000015404

2. 4 months

12. Melino S, Leo S, Toska Papajani V. Natural Hydrogen Sulfide Donors from Allium sp. as a Nutraceutical Approach in Type 2 Diabetes Prevention and Therapy. Nutrients. 2019;11, 10.3390/nu11071581

1. Review

13. Lafuente M, Ortin L, Argente M, Guindo JL, Lopez-Bernal MD, Lopez-Roman FJ, et al. Three-Year Outcomes in a Randomized Single-Blind Controlled Trial of Intravitreal Ranibizumab and Oral Supplementation with Docosahexaenoic Acid and Antioxidants for Diabetic Macular Edema. Retina. 2019;39, 1083-90. 10.1097/IAE.0000000000002114

Included

14. Sepahi S, Mohajeri SA, Hosseini SM, Khodaverdi E, Shoeibi N, Namdari M, et al. Effects of Crocin on Diabetic Maculopathy: A Placebo-Controlled Randomized Clinical Trial. Am J Ophthalmol. 2018;190, 89-98. 10.1016/j.ajo.2018.03.007

Included

15. Neelam K, Goenadi CJ, Lun K, Yip CC, Au Eong KG. Putative protective role of lutein and zeaxanthin in diabetic retinopathy. Br J Ophthalmol. 2017;101, 551-8. 10.1136/bjophthalmol-2016-309814

1. Review

16. Laubertova L, Konarikova K, Gbelcova H, Durackova Z, Muchova J, Garaiova I, et al. Fish oil emulsion supplementation might improve quality of life of diabetic patients due to its antioxidant and anti-inflammatory properties. Nutr Res. 2017;46, 49-58. 10.1016/j.nutres.2017.07.012

1. In vitro study

17. Lafuente M, Ortin L, Argente M, Guindo JL, Lopez-Bernal MD, Lopez-Roman FJ, et al. COMBINED INTRAVITREAL RANIBIZUMAB AND ORAL SUPPLEMENTATION WITH DOCOSAHEXAENOIC ACID AND ANTIOXIDANTS FOR DIABETIC MACULAR EDEMA: Two-Year Randomized Single-Blind Controlled Trial Results. Retina. 2017;37, 1277-86. 10.1097/IAE.0000000000001363

Included

18. Jia YP, Sun L, Yu HS, Liang LP, Li W, Ding H, et al. The Pharmacological Effects of Lutein and Zeaxanthin on Visual Disorders and Cognition Diseases. Molecules. 2017;22, 10.3390/molecules22040610

1. Review

19. Nataraj J, Manivasagam T, Thenmozhi AJ, Essa MM. Lutein protects dopaminergic neurons against MPTP-induced apoptotic death and motor dysfunction by ameliorating mitochondrial disruption and oxidative stress. Nutr Neurosci. 2016;19, 237-46. 10.1179/1476830515Y.0000000010

1. Animal study

20. Mitra RN, Conley SM, Naash MI. Therapeutic Approach of Nanotechnology for Oxidative Stress Induced Ocular Neurodegenerative Diseases. Adv Exp Med Biol. 2016;854, 463-9. 10.1007/978-3-319-17121-0_62

1 Review

21. Ghoneim MAM, Hassan AI, Mahmoud MG, Asker MS. Effect of polysaccharide from Bacillus subtilis sp. on cardio-vascular diseases and atherogenic indices in diabetic rats. BMC Complement Altern Med. 2016;16, 112. 10.1186/s12906-016-1093-1

1. Animal study

22. Chous AP, Richer SP, Gerson JD, Kowluru RA. The Diabetes Visual Function Supplement Study (DiVFuSS). Br J Ophthalmol. 2016;100, 227-34. 10.1136/bjophthalmol-2014-306534

Included

23. Abu-Amero KK, Kondkar AA, Chalam KV. Resveratrol and Ophthalmic Diseases. Nutrients. 2016;8, 200. 10.3390/nu8040200

1. Review

24. Roig-Revert MJ, Lleo-Perez A, Zanon-Moreno V, Vivar-Llopis B, Marin-Montiel J, Dolz-Marco R, et al. Enhanced Oxidative Stress and Other Potential Biomarkers for Retinopathy in Type 2 Diabetics: Beneficial Effects of the Nutraceutic Supplements. Biomed Res Int. 2015;2015, 408180. 10.1155/2015/408180

Included

25. Ghadiri Soufi F, Arbabi-Aval E, Rezaei Kanavi M, Ahmadieh H. Anti-inflammatory properties of resveratrol in the retinas of type 2 diabetic rats. Clin Exp Pharmacol Physiol. 2015;42, 63-8. 10.1111/1440-1681.12326

1. Animal study

26. Domanico D, Fragiotta S, Cutini A, Carnevale C, Zompatori L, Vingolo EM. Circulating levels of reactive oxygen species in patients with nonproliferative diabetic retinopathy and the influence of antioxidant supplementation: 6-month follow-up. Indian J Ophthalmol. 2015;63, 9-14. 10.4103/0301-4738.151455

Included

27. Shin JY, Yu HG. Chlorogenic acid supplementation improves multifocal electroretinography in patients with retinitis pigmentosa. J Korean Med Sci. 2014;29, 117-21. 10.3346/jkms.2014.29.1.117

2. Retinitis pigmentosa

28. Pinazo-Duran MD, Gallego-Pinazo R, Garcia-Medina JJ, Zanon-Moreno V, Nucci C, Dolz-Marco R, et al. Oxidative stress and its downstream signaling in aging eyes. Clin Interv Aging. 2014;9, 637-52. 10.2147/CIA.S52662

1. Review

29. Michan S, Juan AM, Hurst CG, Cui Z, Evans LP, Hatton CJ, et al. Sirtuin1 over-expression does not impact retinal vascular and neuronal degeneration in a mouse model of oxygen-induced retinopathy. PLoS One. 2014;9, e85031. 10.1371/journal.pone.0085031

1. Animal study

30. Grover AK, Samson SE. Antioxidants and vision health: facts and fiction. Mol Cell Biochem. 2014;388, 173-83. 10.1007/s11010-013-1908-z

1. Review

31. Gao W, Li X, Gao Z, Li H. Iron increases diabetes-induced kidney injury and oxidative stress in rats. Biol Trace Elem Res. 2014;160, 368-75. 10.1007/s12011-014-0021-9

1. Animal study

32. Bola C, Bartlett H, Eperjesi F. Resveratrol and the eye: activity and molecular mechanisms. Graefes Arch Clin Exp Ophthalmol. 2014;252, 699-713. 10.1007/s00417-014-2604-8

1. Review

33. Longo-Mbenza B, Muaka M. The protective effect of phaseolus vulgaris on cataract in type 2 diabetes: a profitable hypothesis. Med Hypothesis Discov Innov Ophthalmol. 2013;2, 105-8.

1. Review

34. Soufi FG, Mohammad-Nejad D, Ahmadieh H. Resveratrol improves diabetic retinopathy possibly through oxidative stress - nuclear factor kappaB - apoptosis pathway. Pharmacol Rep. 2012;64, 1505-14. 10.1016/s1734-1140(12)70948-9

1. Animal model

35. Wilkinson JT, Fraunfelder FW. Use of herbal medicines and nutritional supplements in ocular disorders: an evidence-based review. Drugs. 2011;71, 2421-34. 10.2165/11596840-000000000-00000

1. Review

36. Garcia-Medina JJ, Pinazo-Duran MD, Garcia-Medina M, Zanon-Moreno V, Pons-Vazquez S. A 5-year follow-up of antioxidant supplementation in type 2 diabetic retimnopathy. Eur J Ophthalmol. 2011;21, 637-43. 10.5301/EJO.2010.6212

Included

37. Sasaki M, Ozawa Y, Kurihara T, Kubota S, Yuki K, Noda K, et al. Neurodegenerative influence of oxidative stress in the retina of a murine model of diabetes. Diabetologia. 2010;53, 971-9. 10.1007/s00125-009-1655-6

1. Animal study

38. Singh PP, Mahadi F, Roy A, Sharma P. Reactive oxygen species, reactive nitrogen species and antioxidants in etiopathogenesis of diabetes mellitus type-2. Indian J Clin Biochem. 2009;24, 324-42. 10.1007/s12291-009-0062-6

1. Review

39. Bartlett HE, Eperjesi F. Nutritional supplementation for type 2 diabetes: a systematic review. Ophthalmic Physiol Opt. 2008;28, 503-23. 10.1111/j.1475-1313.2008.00595.x

1. Review

40. Nakagawa K, Shibata A, Yamashita S, Tsuzuki T, Kariya J, Oikawa S, et al. In vivo angiogenesis is suppressed by unsaturated vitamin E, tocotrienol. J Nutr. 2007;137, 1938-43. 10.1093/jn/137.8.1938

1. Animal model

41. Cangemi FE. TOZAL Study: an open case control study of an oral antioxidant and omega-3 supplement for dry AMD. BMC Ophthalmol. 2007;7, 3. 10.1186/1471-2415-7-3

2. Age-related macular disease

42. Oh HY, Kim SS, Chung HY, Yoon S. Isoflavone supplements exert hormonal and antioxidant effects in postmenopausal Korean women with diabetic retinopathy. J Med Food. 2005;8, 1-7. 10.1089/jmf.2005.8.1

3 and 4. 12 patients 12 weeks

43. McCarty MF. The putative therapeutic value of high-dose selenium in proliferative retinopathies may reflect down-regulation of VEGF production by the hypoxic retina. Med Hypotheses. 2005;64, 159-61. 10.1016/j.mehy.2002.11.003

1. Review

44. Millen AE, Klein R, Folsom AR, Stevens J, Palta M, Mares JA. Relation between intake of vitamins C and E and risk of diabetic retinopathy in the Atherosclerosis Risk in Communities Study. Am J Clin Nutr. 2004;79, 865-73. 10.1093/ajcn/79.5.865

1. Observational study

45. Millen AE, Gruber M, Klein R, Klein BE, Palta M, Mares JA. Relations of serum ascorbic acid and alpha-tocopherol to diabetic retinopathy in the Third National Health and Nutrition Examination Survey. Am J Epidemiol. 2003;158, 225-33. 10.1093/aje/kwg116

1. Observational study

46. Di Leo MA, Ghirlanda G, Gentiloni Silveri N, Giardina B, Franconi F, Santini SA. Potential therapeutic effect of anti-oxidants in experimental diabetic retina: a comparison between chronic taurine and vitamin E plus selenium supplementations. Free Radic Res. 2003;37, 323-30. 10.1080/1071576021000055271

1. Animal model

47. Hagberg L. Eating for better vision and healthy eyes. Diabetes Self Manag. 2002;19, 8, 10, 2-3.

1. Review

48. Kowluru RA, Tang J, Kern TS. Abnormalities of retinal metabolism in diabetes and experimental galactosemia. VII. Effect of long-term administration of antioxidants on the development of retinopathy. Diabetes. 2001;50, 1938-42. 10.2337/diabetes.50.8.1938

1. Animal model

49. Bursell SE, Clermont AC, Aiello LP, Aiello LM, Schlossman DK, Feener EP, et al. High-dose vitamin E supplementation normalizes retinal blood flow and creatinine clearance in patients with type 1 diabetes. Diabetes Care. 1999;22, 1245-51. 10.2337/diacare.22.8.1245

Included

50. Mayer-Davis EJ, Bell RA, Reboussin BA, Rushing J, Marshall JA, Hamman RF. Antioxidant nutrient intake and diabetic retinopathy: the San Luis Valley Diabetes Study. Ophthalmology. 1998;105, 2264-70. 10.1016/S0161-6420(98)91227-1

1. Review

<u>5. “Extract” (abstract) AND “diabetic retinopathy” (abstract) AND “trial” (abstract)</u>

1. Moon SW, Shin YU, Cho H, Bae SH, Kim HK, and for the Mogen Study G. Effect of grape seed proanthocyanidin extract on hard exudates in patients with non-proliferative diabetic retinopathy. Medicine (Baltimore). 2019;98, e15515. 10.1097/MD.0000000000015515

Included

2. Thiraphatthanavong P, Wattanathorn J, Muchimapura S, Thukham-mee W, Lertrat K, Suriharn B. The combined extract of purple waxy corn and ginger prevents cataractogenesis and retinopathy in streptozotocin-diabetic rats. Oxid Med Cell Longev. 2014;2014, 789406. 10.1155/2014/789406

1. Animal model

3.Watanabe K, Shimada A, Miyaki K, Hirakata A, Matsuoka K, Omae K, et al. Long-term effects of goshajinkigan in prevention of diabetic complications: a randomized open-labeled clinical trial. Evid Based Complement Alternat Med. 2014;2014, 128726. 10.1155/2014/128726

Included. No DR patients initially included but its development was analyzed.

4.Bernardczyk-Meller J, Siwiec-Proscinska J, Stankiewicz W, Fichna P, Pecold K, Korman E. [Influence of Eqb 761 on the function of the retina in children and adolescent with long lasting diabetes mellitus--preliminary report]. Klin Oczna. 2004;106, 569-71.

5. Article in Polish

5. Lanthony P, Cosson JP. [The course of color vision in early diabetic retinopathy treated with Ginkgo biloba extract. A preliminary double-blind versus placebo study]. J Fr Ophtalmol. 1988;11, 671-4.

4. Inclusion of 29 patients
